# Cryo‐EM Structures Reveal the Molecular Basis of Asymmetric Allosteric Activation by MMOB in the Hydroxylase of Soluble Methane Monooxygenase

**DOI:** 10.1002/advs.202517312

**Published:** 2026-01-21

**Authors:** Yunha Hwang, Bumhan Ryu, Soyeon Park, Dong‐Heon Lee, Hyo Jin Hong, Jeong‐Geol Na, Chul Gyu Song, Hyun Goo Kang, Edwin Pozharski, Seung Jae Lee

**Affiliations:** ^1^ Department of Chemistry Jeonbuk National University Jeonju Republic of Korea; ^2^ Research Solution Center Institute For Basic Science Daejeon Republic of Korea; ^3^ Department of Chemical Biomolecular Engineering Sogang University Seoul Republic of Korea; ^4^ Institute for ICT‐Based Infectious Disease Technology Research and Department of Electronic Engineering Jeonju Republic of Korea; ^5^ Department of Neurology and Research Institute of Clinical Medicine Jeonbuk National University Jeonju Republic of Korea; ^6^ Institute for Biochemistry and Molecular Biology School of Medicine University of Maryland Baltimore Maryland USA; ^7^ Institute for Bioscience and Biotechnology Research University of Maryland Rockville Maryland USA; ^8^ Research Institute For Molecular Biology and Genetics Jeonbuk National University Jeonju Republic of Korea

**Keywords:** allosteric effect, cryogenic electron microscopy, di‐iron active site, greenhouse gas, methane monooxygenase

## Abstract

Soluble methane monooxygenase (sMMO) catalyzes the hydroxylation of methane at non‐heme di‐iron active sites under ambient conditions. The regulatory component (MMOB) is essential for catalytic activity, inducing conformational changes in the active site and facilitating substrate ingress in hydroxylase (MMOH). Advances in cryogenic electron microscopy (cryo‐EM) have enabled structural studies of sMMO under near‐native conditions. 3D variability analysis reveals that an asymmetric MMOH–MMOB complex predominates in solution, supporting a sequential binding mechanism. Here, we report a 2.85 Å‐resolution cryo‐EM structure of MMOH–1MMOB (H‐1B) complex, in which a single MMOB binds to MMOH and generates two distinct protomers: MMOB‐bound protomer (HB^A^, αβγB) and non‐MMOB‐bound protomer (HB^B^, αβγ). MMOB initiates an allosteric cascade beginning at the *N*‐terminal region of the HB^A^ β‐subunit and extending to the di‐iron active site. This structural shift shortens the Fe···Fe distance in HB^A^ to 2.7 Å, consistent with a geometry conducive to O_2_ activation, while HB^B^ retains a 3.1 Å distance. The γ‐subunit modulates this asymmetry by stabilizing the resting HB^B^ and facilitating the reorganization of HB^A^. These findings support an asymmetric catalytic cycle that allows continuous hydroxylation and promotes electron transfer, thereby providing a structural basis for future mechanistic studies.

## Graphical abstract

Cryo‐EM analysis reveals an asymmetric MMOH–MMOB complex with distinct protomers, where MMOB binding triggers structural rearrangements from the surface to the active site. This conformational asymmetry shortens the Fe···Fe distance to 2.7 Å in one protomer, forming a geometry suitable for O_2_ activation and supporting a sequential catalytic mechanism.

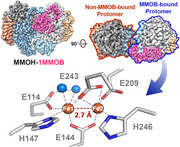

## 1 Introduction

Methane (CH_4_) is a potent greenhouse gas with a global warming potential (GWP) approximately 83 times higher than that of carbon dioxide (CO_2_) over the past 20 years, despite its lower atmospheric concentration [1, 2, 3]. The C─H bond in methane exhibits a high bond dissociation energy (∼104.9 kcal/mol), which hinders its activation under ambient conditions [4, 5, 6]. Methane monooxygenases (MMOs) from methanotrophic bacteria, including soluble MMO (sMMO) and particulate MMO (pMMO), can convert methane to methanol under atmospheric conditions [7, 8, 9]. Understanding the mechanisms by which MMOs activate methane can provide a basis for developing catalysts [10, 11, 12]. sMMO belongs to the bacterial multi‐component monooxygenase (BMM) superfamily, which includes the key hydroxylase (MMOH), regulatory component (MMOB), and reductase (MMOR) [13, 14, 15]. Crystal structures of MMOH, MMOH–MMOB, and MMOH–MMOD (inhibitory component) complexes have been reported, providing a foundation for mechanistic studies of O_2_ intermediates and C─H activation [16, 17, 18, 19]. MMOH forms a homodimer composed of two αβγ protomers, predominantly α‐helices in structure [20, 21]. The α‐subunit contains the non‐heme di‐iron active site responsible for C─H activation, whereas the roles of the β‐ and γ‐subunits remain unclear [22, 23, 24]. The dimeric interface of MMOH, known as the “canyon region,” serves as the binding site for auxiliary components such as MMOB and MMOD [17, 19]. Interactions with these components modulate the coordination geometry of the di‐iron center and support substrate delivery pathways, thereby accelerating or decelerating the methane hydroxylation [17, 18, 19].

The crystallographic structures of MMOH and its complexes have enhanced our understanding of enzyme reactions but may not fully reflect the native and dynamic behavior of sMMO due to lattice‐imposed symmetry and static conformation. This limitation underscores the need for structural approaches to elucidate regulatory mechanisms near physiological conditions. Recent advances in cryogenic electron microscopy (cryo‐EM) have opened avenues for high‐resolution structural analysis of proteins, driven by improvements in both hardware and computational algorithms [25, 26, 27]. The vitrification process, involving rapid freezing, preserves proteins in their native states without requiring crystallization, unlike X‐ray crystallography [28, 29]. Furthermore, cryo‐EM provides insights into conformational dynamics by capturing multiple structural states within a single dataset [29, 30, 31]. Computational approaches such as 3D variability analysis (3DVA) and 3D flexible refinement (3DFlex) resolve continuous structural heterogeneity and flexible motions in macromolecular assemblies [31, 32].

MMOH represents an ideal target for cryo‐EM analysis due to its high α‐helical content and dynamic interactions with a range of auxiliary components, including MMOB, MMOR, MMOD, and MMOG (chaperone) [33, 34, 35]. Reported crystal structures of sMMO complexes have revealed only symmetric assemblies, with identical components bound to both canyon regions [17, 18, 19]. The observed symmetry, imposed by crystal packing, may provide only a limited perspective on the regulatory interactions. Cryo‐EM allows visualization of sMMO complexes under near‐native conditions, allowing researchers to observe structural variability and dynamic associations with auxiliary components [31, 32]. This structural heterogeneity offers valuable insights into the geometric arrangement of the di‐iron active site and supports refined computational modeling of the catalytic pathway. The structural asymmetry observed in cryo‐EM may facilitate interactions with auxiliary components, such as MMOR, during electron transfer.

Here, we report the first cryo‐EM structure of an asymmetric MMOH–1MMOB (H‐1B) complex from *Methylosinus sporium* 5 (*M. sporium* 5), captured for atomic analysis. It represents the solution structure. Results demonstrate a predominant H‐1B configuration, in which a single MMOB binds asymmetrically to the MMOH dimer. This asymmetry permits the direct comparison of active and resting protomers within the same molecule and reveals structural rearrangements for efficient C─H or O_2_ activation, triggered at the β‐subunit toward the di‐iron center. The γ‐subunit, previously considered a structural scaffold, plays an active role in coordinating the asymmetric rearrangements. These observations demonstrate a mechanistic framework in which MMOB orchestrates substrate access, redox‐state modulation, and first‐sphere rearrangement at the di‐iron site essential for sMMO catalysis.

## 2 Results and Discussion

### 2.1 Dynamics of MMOH–1MMOB Complex at Near‐Native State

**FIGURE 1:**
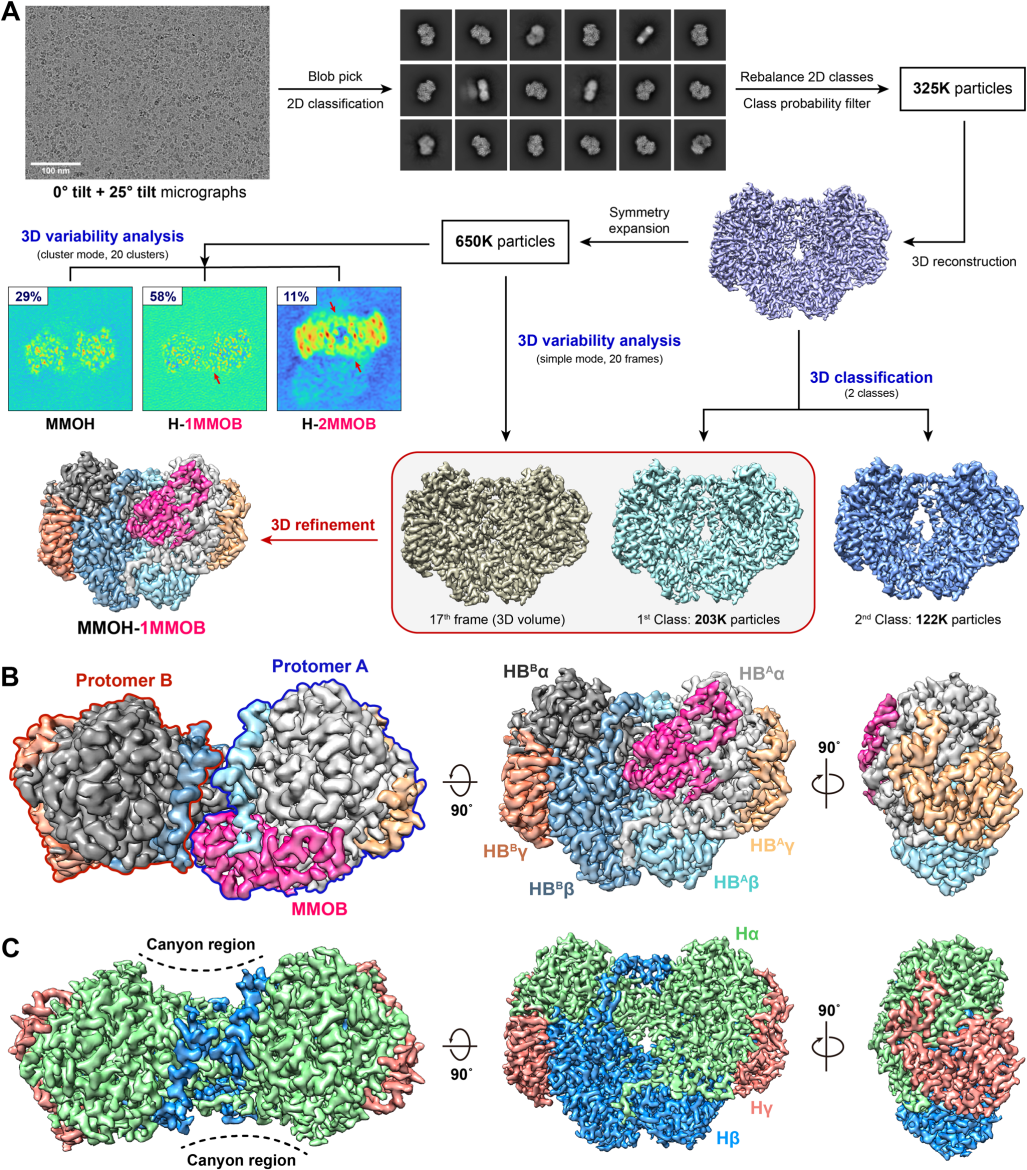
Complex formation of MMOH and MMOB in the near‐native state. (A) Flow chart depicting the cryo‐EM image processing pipeline for the MMOH–MMOB complex (H‐1B, PDB: 8XIW). ‘K’ denotes the number of particles in thousands. Red arrows depict MMOB using 3DVA in cluster mode. (B) Top, front, and side views of the H‐1B complex. MMOB (hot pink) binds to the protomer A (α‐subunit, gray; β‐subunit, aquamarine; γ‐subunit, bright orange) of MMOH (HB^A^). (C) MMOH in native state with cryo‐EM (PDB: 8YRD). Top, front, and side views of MMOH (α‐subunit, lime; β‐subunit, marine; γ‐subunit, deep salmon).

Structural elucidation of MMOH using cryo‐EM is hindered by its preferential front‐view orientation in vitreous ice [36, 37, 38], which limits the reconstruction of a 3D map despite its relatively large molecular weight (>248 kDa) [39]. A recent cryo‐EM study has reported a high‐resolution structure of the MMOH from *Methylococcus capsulatus* Bath (*M. capsulatus* Bath) using graphene grids [40], noting that orientation preference must be addressed to enable robust 3D reconstruction. Employment of complementary approaches overcomes this hurdle through stage tilting and 2D‐rebalancing [41, 42], which reduce orientation bias and improve angular distribution (Figure 1A; Figure S1). These strategies enable the reconstruction of high‐resolution (∼2.7 Å) MMOH–MMOB complex, although MMOB density is only detectable at a low contour level (data not shown). The contour level represents the density threshold used for visualization in cryo‐EM maps [43, 44], and the weak appearance of MMOB at higher thresholds suggests low occupancy or decreased signal. This weak MMOB signal may result from the imposition of C_2_ symmetry during reconstruction, based on previous crystallographic structures showing two MMOB molecules symmetrically bound to MMOH (H‐2B) [17, 18]. We apply 3DVA to classify particles into discrete structural intermediates, thereby representing the conformational heterogeneity of the MMOH–MMOB complex (Figure 1A). This analysis indicates that the majority of particles (58%) represent an asymmetric H‐1B configuration, in which a single MMOB molecule binds to MMOH. The predominance of this state suggests that the H‐1B configuration is a reasonable candidate for the catalytically relevant sMMO structure in the solution. The H‐2B configuration is also observed with 11% occupancy (Figure S1), although this assembly appears to result from the linear stacking of two H‐2B molecules (Figure S2A,B). The 3D reconstruction of the stacked H‐2B particles shows a packing arrangement that resembles the crystallographic lattice observed in previously reported structures (Figure S2C,D). This finding suggests that the symmetric H‐2B form may represent an energetically stabilized under crystallization or particle stacking conditions, although we cannot rule out the possibility that the symmetric H‐2B can contribute to catalytic activities [45]. A homogeneous set of H‐1B particles is obtained by excluding stacked H‐2B particles through 2D and 3D classification (Figure S1), which allows high‐resolution reconstruction of the MMOH–MMOB complex in its native asymmetric configuration.

High‐resolution structural determination of the H‐1B complex requires a volume that reflects its most representative conformational state, due to the flexibility of MMOH and its dynamic interaction with MMOB. 3DVA is further applied to capture flexible and transient motions of the MMOH–MMOB complex by showing continuous conformational changes (Videos S1–S3). This analysis demonstrates the first sequential binding of MMOB to the canyon regions of MMOH, showing that early frames (first–8^th^) capture MMOB release from one canyon, whereas later frames (nineth–20^th^) depict MMOB binding at the opposite canyon in MMOH. The 17^th^ frame is identified as the most representative MMOB‐bound conformation and is selected as the reference volume for high‐resolution refinement (Figure 1A). The cryo‐EM structure of the H–1B complex is determined at 2.85 Å resolution (PDB: 8XIW; Figure 1B and Table S1), indicating a stoichiometry in which a single MMOB molecule binds asymmetrically to one protomer of MMOH. This asymmetric configuration has not been observed in previous X‐ray diffraction or X‐ray free electron laser (XFEL) studies [17, 18]. We consider that the MMOB‐bound protomer (HB^A^, αβγB) represents an active state, while the non‐MMOB‐bound protomer (HB^B^, αβγ) remains in resting state (Figure 1B). This asymmetry implies that the two protomers exhibit distinct functional roles during the catalytic cycle. The HB^A^ protomer adopts the configuration associated with methane hydroxylation, whereas the HB^B^ protomer assumes a resting arrangement positioned to engage auxiliary components such as MMOR. This functional asymmetry provides a structural basis for understanding how sMMO coordinates electron transfer and substrate activation within its dimeric assembly (vide infra).

A cryo‐EM structure of MMOH alone can provide valuable information to analyze MMOB‐induced conformational changes by comparison with the H‐1B structure. The 3D classification separates two distinct conformations based on molecular flexibility upon rapid freezing (Figure S3), enabling the elucidation of a 2.64 Å‐resolution cryo‐EM structure of MMOH (PDB: 8YRD; Figure 1C; Table S1). MMOH contains multiple long α‐helices across its three subunits (α, β, and γ), and the helix nomenclature used in this study follows the system established in the first X‐ray structure of MMOH [16]. That study denoted the long helices in the α‐subunit by letters (A–H) and short helices by numbers, whereas helices in the γ‐subunit were numbered separately [16]. We applied an α‐prefix to the γ‐subunit helix numbers to distinguish them from α‐subunit helices in our cryo‐EM structures (Figure S4). Structural analysis demonstrates the protein dynamics of the heart‐shaped MMOH molecule, which exhibits a ‘beating heart’ type motion (Videos S4–S6), although its translational shifts are weak compared to the H‐1B complex (Videos S1–S3). This structure serves as a reference for explaining the conformational changes induced by MMOB and for differentiating them from the intrinsic dynamics of MMOH.

### 2.2 MMOB‐Driven Allosteric Effects From Surface to Di‐Iron Active Site

**FIGURE 2:**
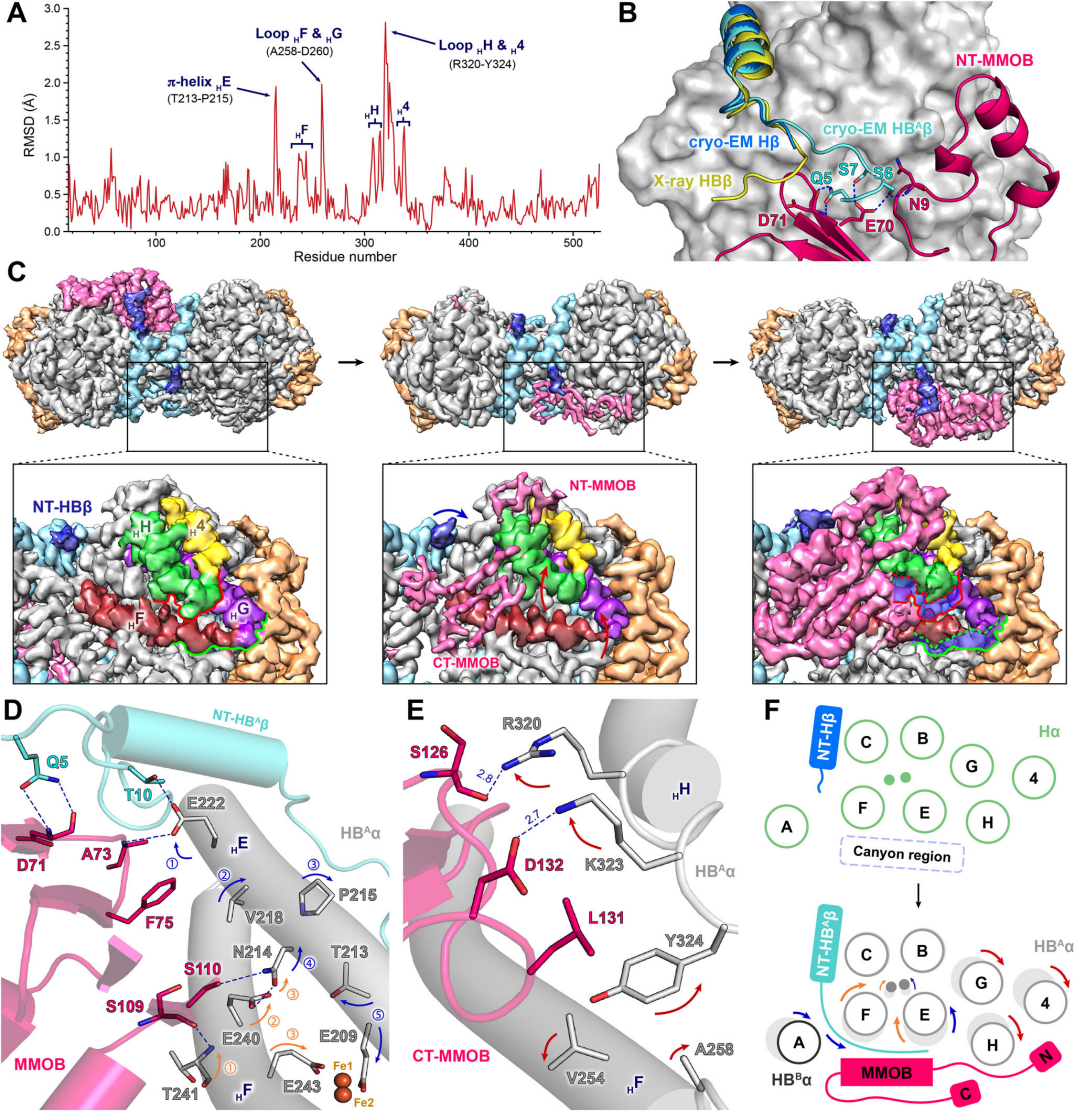
Conformational changes in MMOH induced by MMOB binding. (A) Root‐mean‐square deviation (RMSD) of the main chain (C_α_) per residue for the α‐subunit of cryo‐EM MMOH and HB^A^. (B) Structural alignment of NT‐MMOHβ from cryo‐EM MMOH, cryo‐EM HB^A^, and X‐ray HB (PDB: 4GAM). Cryo‐EM HB^A^α (gray) is shown using a surface representation model. (C) Molecular motions of MMOH and MMOB captured by 3DVA in the simple mode (fourth, nineth, and 18^th^ frame). The subscript “H” before an English letter denotes a helix. In the fourth frame, solid lines represent the initial positions of helices F, G, H, and 4, which undergo shifts upon MMOB binding and reach their final positions as indicated by dashed lines of the same color in the 18^th^ frame. (D) NT‐HB^A^β and MMOB affect the di‐iron active site by inducing shifts in helices E and F of HB^A^α. (E) CT‐MMOB induces a shift in helix H of HB^A^α. The color of the arrows indicates conformational changes caused by CT‐MMOB (red), NT‐HB^A^β (blue), and the core region of MMOB (orange). The unit of distance is Ångström (Å). (F) Schematic model illustrating the allosteric regulation of MMOH via its interaction with MMOB.

Reported crystallographic studies are limited in explaining the dynamic roles of the β‐ and γ‐subunits due to lattice‐imposed symmetry [16, 17, 18, 19], which have limitations to elucidate the mechanism of the catalytic cycle. The MMOH–MMOD complex proposed that the long α‐helix (Ser51–Ala72) of MMOD disrupts the *N*‐terminal region of the MMOH β‐subunit (NT‐Hβ), leading to rearrangement of the four‐helix bundle, including helices B, C, E, and F, and the di‐iron active site [19]. The regulatory role of NT‐Hβ in MMOB‐mediated activation has remained unclear, however. The cryo‐EM structure of the asymmetric H‐1B complex allows comparison between HB^A^ and HB^B^ within the same molecule (Figure 1B). Root‐mean‐square deviation (RMSD) analysis confirms significant conformational differences in the α‐subunit between MMOH alone and HB^A^ from the cryo‐EM (Figure 2A). The comparison between HB^A^ and MMOH alone provides direct structural evidence for explaining MMOB‐induced changes associated with catalytic activity. Comparison between HB^A^ and HB^B^ protomers further demonstrates the asymmetric nature of these conformational changes within the H‐1B complex (Figure S5). The flexible *N*‐terminal region of the HB^A^ β‐subunit (NT‐HB^A^β; Pro4–Arg12) becomes rigidified upon MMOB binding (Figure 2B; Figure S6). This short loop interacts with MMOB, accounting for 25% (5 out of 20) of all hydrogen bonds at the MMOH–MMOB interface (Figure S6D), implying its critical role in mediating allosteric activation. 3DVA revealed that MMOB induces pronounced conformational changes in NT‐HB^A^β, a region previously unrecognized as a key contributor to the allosteric regulation of MMOH, with the fourth, nineth, and 18^th^ frames providing representative snapshots of this sequential binding (Figure 2C; Videos S1–S3). The structural transition of NT‐HB^A^β not only stabilizes helices B and C but also initiates a cascade of conformational shifts toward the di‐iron center (Figure S7). Gln5 of HB^A^β forms a hydrogen bond with Asp71 of MMOB, which also interacts with Glu222 of HB^A^α (Figure 2D and Figure S6D). These interactions induce a positional shift of the four‐helix bundle, thereby altering the geometry of key MMOHα residues at the active site, such as Glu243 and Glu209, for di‐iron coordination (Figure 2D; Figure S8A,B). The observed coupling between NT‐HB^A^β and the four‐helix bundle suggests that NT‐HB^A^β functions as an allosteric trigger for C─H activation. Quantum mechanics/molecular mechanics (QM/MM) simulations have been widely used to characterize the electronic and geometric properties of the catalytically active di‐iron environment [46, 47]. Results from these approaches, however, did not consider the long‐range allosteric effects originating from NT‐HB^A^β, as reported by our cryo‐EM H‐1B. These considerations for the di‐iron site will be a critical framework for refining future QM/MM simulations and advancing mechanistic insights into sMMO mechanisms.

Helices F, G, H, and 4 of the α‐subunit undergo conformational rearrangements upon MMOB binding (Figure 2C and Videos S1–S3). These structural changes arise from both the allosteric influence initiated at NT‐HB^A^β and the interactions involving the *N*‐ and *C*‐terminal regions of MMOB (Figure S6A,B). Crystallographic studies have previously shown that the *N*‐terminal region of MMOB (NT‐MMOB) generates an α‐helical conformation that anchors MMOB within the canyon of MMOH [17]. Previous biochemical and mutagenesis studies have shown that truncations or substitutions within the NT‐MMOB diminish sMMO activity, underscoring its essential role in catalysis [48, 49, 50]. The H‐1B complex shows that NT‐MMOB reinforces this anchoring role through a hydrogen bond formed between Glu27 of MMOB and Arg333 of helix 4 in HB^A^α (Figure S6E–G). Earlier mutagenesis studies have supported the catalytic importance of the *C*‐terminal region of MMOB (CT‐MMOB), although its structural contribution had remained uncharacterized [51, 52]. Truncation mutants of CT‐MMOB have shown that the deletion of residues, Ala134–Val139, retained ∼90% of wild‐type activity, while deletion extending to Leu131 and Asp132 reduced activity to 63%; removal of Ser126 (Δ126–139) further decreased activity to 28% [51]. The H‐1B structure now reveals specific interactions between CT‐MMOB and helices H and F of HB^A^α (Figure 2C), providing a structural basis for these functional deficits. Ser126 and Asp132 of CT‐MMOB form hydrogen bonds with Arg320 and Lys323 of helix H, while Leu131 of CT‐MMOB inserts into a hydrophobic pocket formed by the aliphatic and aromatic residues in helices F and H (Figure 2E; Figure S8C,D). These interactions demonstrate that NT‐ and CT‐MMOB drive conformational changes from the surface to the di‐iron active site. The resulting structural rearrangements, initiated at NT‐HB^A^β through helices E, F, G, H, and 4, reorganize the α‐subunit presumably to promote catalytic activity (Figure 2F). Our cryo‐EM analysis thus illustrates the more nearly complete allosteric pathway, providing a previously uncharacterized structural framework to elucidate catalytic mechanisms.

The functional role of the MMOH γ‐subunit (MMOHγ) has remained poorly understood. The cryo‐EM H‐1B complex allows direct comparison of the two MMOHγ chains within the same molecule, enabling assessment of MMOB‐induced asymmetry that was obscured in previous symmetric structures (Figure S9A–C). At the *C*‐terminus of MMOHγ (CT‐Hγ), Arg157 and Arg160 in the α8‐helix form hydrogen bonds with Glu454 and Leu458 of MMOHα, restricting the translational movement of helices G, H, and 4 (Figure S9D). A short random coil (Lys163–Leu167) at the CT‐Hγ aligns with the β3‐strand (R437–F441) of MMOHα (Figure S9D). This coil undergoes a conformational change into a new β‐strand upon MMOB‐induced repositioning of Leu165 (Figure S9E,F). The core helices of MMOHγ (α3, α4, and α7) participate in asymmetric modulation by stabilizing HB^A^ and relaxing HB^B^ via interactions with the flexible loops of MMOHβ (Figure S9G–I). These inter‐subunit interactions are mediated by specific hydrogen bonds, including those formed by Gln55 and Arg116 of MMOHγ with Asp74 and Glu89 of MMOHβ, respectively (Figure S9G). Upon MMOB binding, these hydrogen bonds are stabilized in HB^A^ and disrupted in HB^B^, reflecting the asymmetric allosteric response at the γ‐ and β‐subunit interface (Figure S9H,I). H‐1B demonstrates that the γ‐subunit is not only a structural scaffold but also an active modulator that orchestrates the asymmetric reorganization of the MMOH protomers.

### 2.3 The Active Protomer with 2.7 Å Fe—Fe Distance

The mechanism of sMMO depends on catalytic intermediates, including the Q‐intermediate, which serves as the potent oxidant responsible for methane hydroxylation [53, 54, 55]. This species is suggested to feature a high‐valent di‐iron center in the Fe(IV)–Fe(IV) oxidation state, which facilitates activation of the C─H bond in methane [56, 57, 58]. Early spectroscopic studies, including extended X‐ray absorption fine structure (EXAFS) and resonance Raman spectroscopy, have supported a closed‐core model for the Q‐intermediate [59, 60]. These analyses revealed a short Fe···Fe distance (∼2.5–2.8 Å) and symmetric vibrational modes consistent with a bis‐µ‐oxo diamond core [8, 47, 59, 60]. In addition, recent studies have proposed an open‐core model for the Q‐intermediate [61]. This model represents an elongated Fe···Fe distance (∼3.4 Å), based on High‐energy resolution fluorescence detection (HERFD) X‐ray absorption spectroscopy and QM/MM simulations [46, 62]. These contrasting models are derived from spectroscopic evidence or crystallographic structure, which are limited in capturing dynamic structural transitions. High‐resolution structural characterization under near‐solution conditions is, therefore, essential to elucidate the geometry of catalytically relevant sMMO intermediate.

**FIGURE 3:**
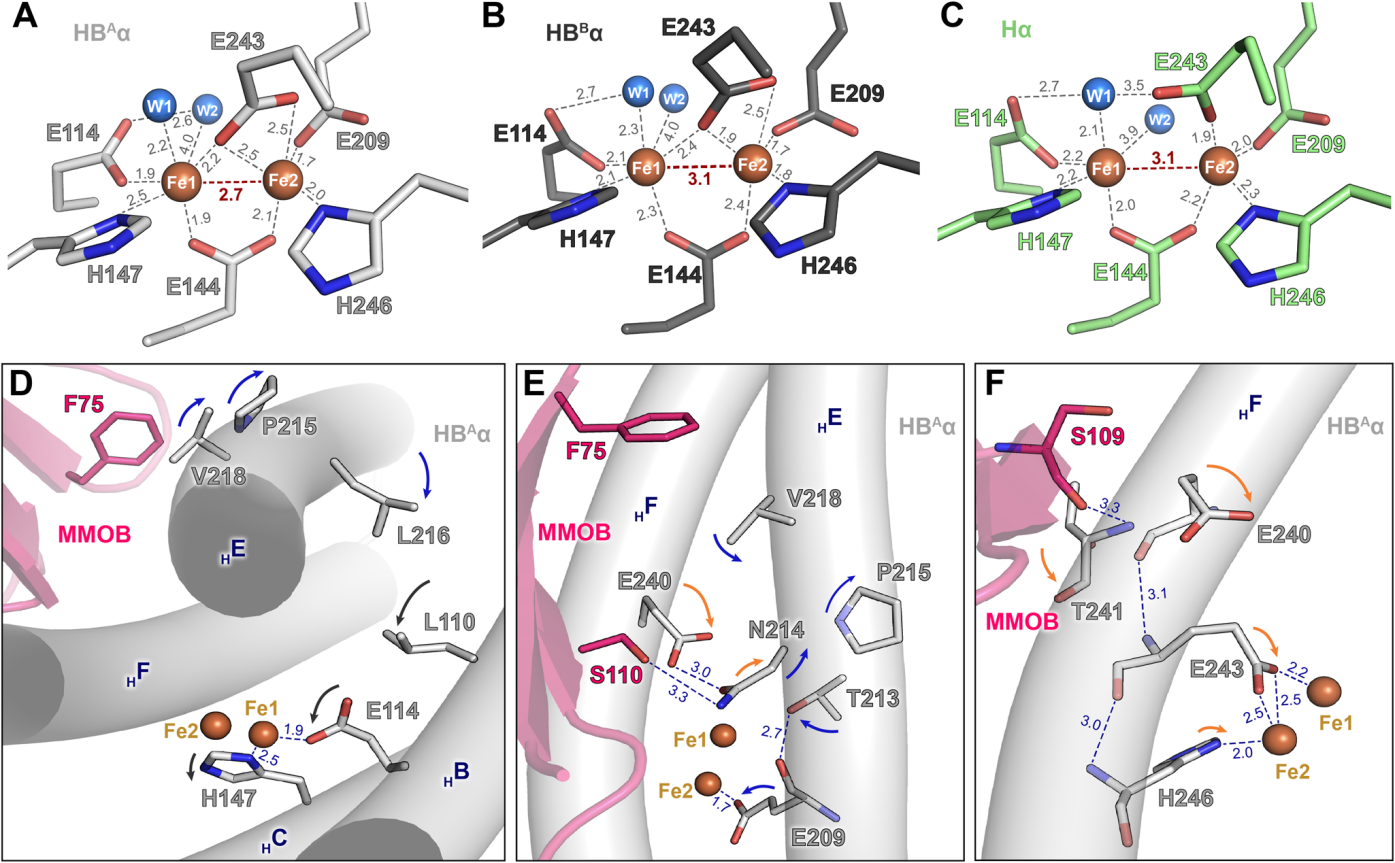
Di‐iron active site in MMOH and H‐1B from cryo‐EM. (A–C) Geometry of the di‐iron center in (A) HB^A^, (B) HB^B^, and (C) MMOH. The two Fe atoms are coordinated by six residues—four glutamates and two histidines. Water molecules are displayed as marine spheres and are labeled W1 and W2 based on their positions. Coordination distances are depicted in gray, while the Fe–Fe distances are represented in red. The unit of distance is Ångström (Å). (D–F) F75, S109, and S110 in MMOB induce the shifting of residues in the four‐helix bundle, which was related to the coordination of di‐iron center. The subscript “H” before an English letter indicates a helix. The color of the arrows indicates conformational changes caused by NT‐HB^A^β (blue) and the core region of MMOB (orange). Black arrows indicate conformational changes in the residues of helices B and C.

Comparison of our cryo‐EM structures with previously reported crystallographic models is supported by the high structural conservation among sMMO species—including *M. sporium* 5, *M. capsulatus* Bath, and *Methylosinus trichosporium* OB3b—which share closely related sequences, folds, and di‐iron active‐site environments [63, 64, 65, 66]. The H‐1B complex shows different coordination at the di‐iron center within the single molecule, representing both the active and resting protomers (Figure 3A,B). This asymmetric configuration reveals distinct geometric features at the di‐iron active site that differ from crystallographic structures (Figures S10 and S11 and Table S2). The cryo‐EM structures exhibit an Fe1···Fe2 distance of 3.1 Å in both MMOH and the resting HB^B^ protomer, consistent with the crystal structure of oxidized MMOH (MMOH_ox_, Fe^3+^–Fe^3+^; Figure S10A,B). The active HB^A^ protomer represents a shortened Fe1···Fe2 distance of 2.7 Å (Figure 3A), suggesting a compressed geometry favorable for O_2_ activation and Q‐intermediate formation. Although the cryo‐EM structure does not represent intermediates, the 2.7 Å Fe···Fe distance observed in HB^A^ reflects a more compact geometry comparable to that proposed for the Q‐intermediate in previous spectroscopic studies [59, 60]. The pre‐compressed arrangement in HB^A^ may favor O_2_ activation by positioning ligands and metal ions in a configuration that is more compatible with subsequent diferryl state formation (Video S7). This structural compression is mainly driven by MMOH‐induced rearrangements around Fe1 (Figure S7). This event caused the shortened bidentate coordination of Glu144 to Fe1 and Fe2, accompanied by elongation of the Fe1–Nδ(His147) distance from 2.2 Å in MMOH to 2.5 Å in HB^A^ (Figure 3A,C). Glu114 changes its position in HB^A^ while remaining coordinated to Fe1; its oxygen atom loses a hydrogen bond with one water molecule (W1) and forms a new hydrogen bond with another water molecule (W2), as described in Figure 3. The compressed geometry of the active site in HB^A^ offers structural insight into a conformation proposed to facilitate Q‐intermediate formation.

Spectroscopic analyses, including circular dichroism (CD), magnetic CD, and ligand field theory, have suggested that MMOB facilitates reduction of the di‐iron active site and promotes O_2_ activation [67]. These studies focused on the reorientation of Glu243 and its impact on Fe2 coordination upon MMOB interaction [23, 67]. Comparison of the cryo‐EM structures between MMOH and the H‐1B complex enables evaluation of the structural basis for MMOB‐induced redox modulation of the di‐iron center (Figure 3A–C). The cryo‐EM MMOH exhibits a di‐ferric coordination consistent with the X‐ray crystallography‐based MMOX_ox_, in which Glu243 was positioned to form a monodentate interaction with Fe2 and a hydrogen bond to W1 (Figure 3C; Figure S10A,B). Glu243 in the HB^A^ protomer of the H‐1B complex coordinates both Fe1 and Fe2 in a bidentate manner (Figure 3A), consistent with the crystal structure of reduced MMOH (MMOH_red_, Fe^2+^–Fe^2+^; Figures S10C,D and Figure S11F). This reconfiguration provides structural insight in which MMOB binding may influence electron transfer to the di‐iron center by inducing a geometry compatible with reduction and catalytic activity. Glu243 in the HB^B^ protomer exhibits a reoriented conformation but remains preferentially coordinated to Fe2, as indicated by its 2.4 Å distance to Fe1 and 1.9 Å to Fe2 (Figure 3B). The continued association of Glu243 to Fe2 and the 3.1 Å Fe1···Fe2 distance indicate that the HB^B^ protomer remains in an oxidized state (Figure 3B,C). This asymmetric coordination environment suggests that MMOB binding selectively induces structural changes in only one protomer, allowing spatial separation of catalytic activation and electron transfer within the dimeric assembly.

The modified coordination at the di‐iron active site can be attributed to conformational changes in second‐ and third‐sphere residues triggered by MMOB, including Phe75, Ser110, and Ser109 (Figure 3D–F). Phe75 (MMOB) induces a positional shift of Val218 (helix E), which initiates a cascade toward Pro215 and Leu216 in helix E, and subsequently affects Leu110 and Glu114 in helix B (Figure 3D; Figure S12A,B). This event rearranges the coordination environment around Fe1 through repositioning of Glu114 and His147, contributing to Fe···Fe compression. Ser110 and Phe75 from MMOB influence residues such as Asn214 and Val218, thereby altering the conformation of helix E (Figure 3E; Figure S12C,D). The resulting conformational shifts modify the position of Thr213 to form hydrogen bonds with Glu209, contributing to the reorganization around Fe2. Ser109 (MMOB) forms a hydrogen bond with Thr241 in helix F, and this further changes the Glu240 conformation (Figure 3F; Figure S12E,F). These consequential effects modify the coordination in Glu243 and His246, where the rotation of Glu243 facilitates its bidentate interaction with the di‐iron center. This structural evidence supports a mechanism for dynamic regulation of sMMO activity within its dimeric assembly in an asymmetric manner.

### 2.4 Reorganization of Hydrophobic Cavities by MMOB for CH_4_ and O_2_ Ingress

**FIGURE 4:**
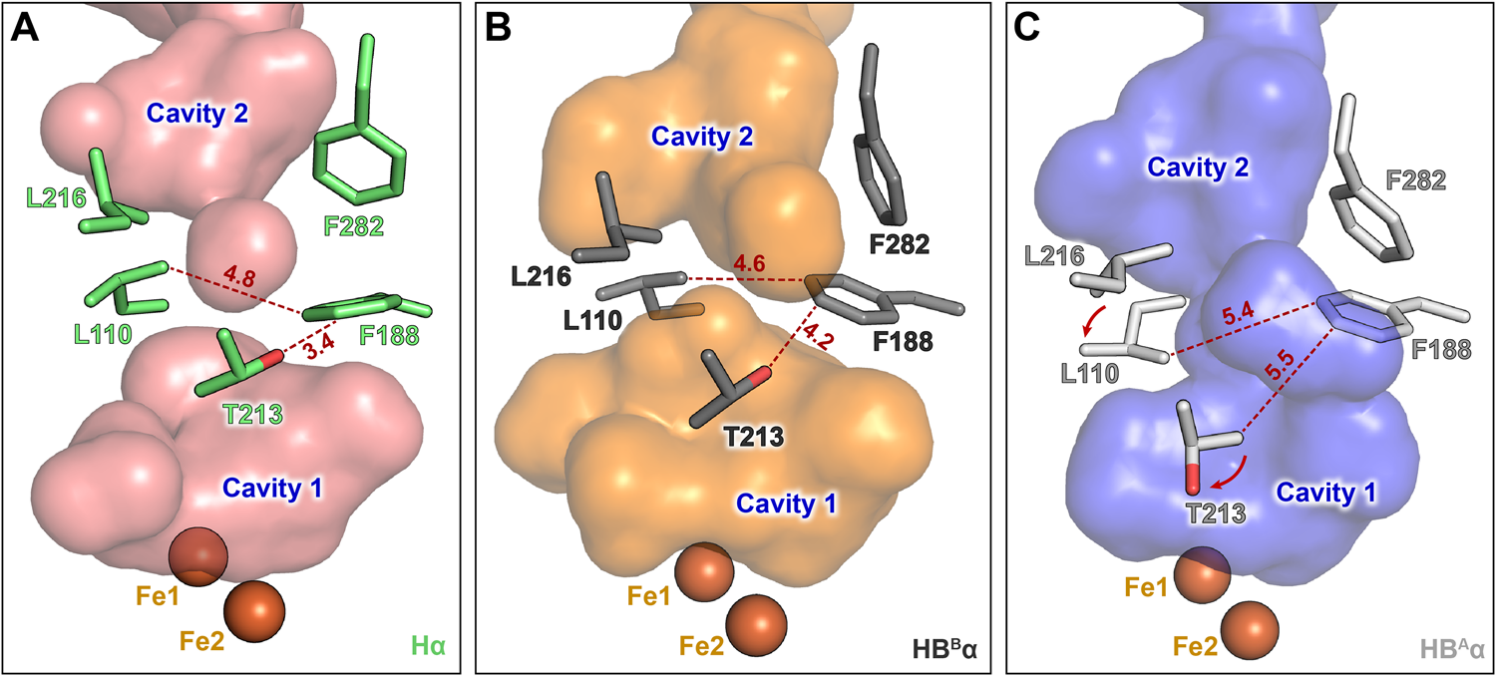
Regulation of the substrate pathway upon MMOB binding to MMOH in the native state. (A–C) Views of cavities 1 and 2 are shown as translucent van der Waals surfaces in the interior of (A) MMOH (residue, lime; surface, deep salmon), (B) HB^B^ (residue, dark; surface, orange), and (C) HB^A^ (residue, gray; surface, tv_blue). The cavities were displayed using PyMOL 2.5.2 are shown as surface models.

Structural analyses of the soluble di‐iron monooxygenase (SDIMO)—including sMMO, phenol hydroxylase (PH), and toluene/o‐xylene monooxygenase (ToMO)—have identified conserved hydrophobic cavities near the di‐iron center that serve as internal pathways for substrate and O_2_ ingress [68, 69, 70, 71]. Three hydrophobic cavities (cavities 1, 2, and 3) were identified in the crystal structure of MMOH_o__x_, where cavities 1 and 2 were disconnected [8, 10]. Previous studies have indicated that Phe188 and Leu110 operate as gatekeepers [17, 33, 72]. Although similar inter‐residue distances (∼6 Å) involving Thr213 and Phe188 are present in earlier MMOH–MMOB crystal structures [17, 18], the cryo‐EM structures elucidate the relevance of Thr213 to the continuity of cavities 1–2 (Video S8). Mutagenesis studies of homologous SDIMOs, including T201 variants of ToMO, have reported that this conserved threonine modulates the accessibility of small molecules to the di‐iron center by altering the reactivity and stabilization of peroxo‐di‐iron intermediates [71, 73]. The T213S mutation in sMMO has been reported to modulate substrate preference, leading to a loss of activity for smaller substrates compared with larger substrates [74]. These prior findings highlight the relevance of Thr213 in regulating internal pathways in sMMO. Our cryo‐EM structures indicate that Thr213 serves as the structural switch that determines the continuity of cavities 1–2, which is revealed by contrasting the two protomers in the asymmetric H‐1B structure (Figure 4; Figure S13A–F). Cryo‐EM structures of MMOH and the HB^B^ protomer reveal that the oxygen atom of Thr213 leads to the collapse of hydrophobic cavities (Figure 4A,B; Figure S13A,B). The rotameric shift of Thr213 in the HB^A^ protomer displaces its polar side chain away from Phe188, increasing the distance to 5.5 Å and thereby creating a continuous hydrophobic cavity toward the di‐iron active site (Figure 4C; Figure S13C). A positional shift of Leu110 away from Phe188 (5.4 Å) further reinforces this opening, contributing to the formation of a continuous substrate‐access cavity (Figure 4C). This structural arrangement is consistent with previous findings in PH and ToMO, where internal hydrophobic channels of ∼6 Å were sufficient to allow the diffusion of small molecules such as O_2_ [13, 71, 75].

MMOB reorients the positions of key hydrophobic residues in the four‐helix bundle, which regulate cavity formation (Figure S13G,H). The connection of these cavities is determined by the movement of Phe75 from MMOB, which induces the rotation of Thr213 in HB^A^ (Figure 3E; Figure S12C,D). This structural shift continues through Val218, Pro215, and Leu216 in helix E, reorienting Leu110 to form a continuous hydrophobic pathway toward the active site (Figure S13G,H). Cavity opening is clearly observed in the HB^A^ protomer, whereas the HB^B^ protomer retains a disconnected cavity similar to MMOH. This structural asymmetry supports an alternating catalytic mechanism, wherein MMOB selectively activates one protomer to couple electron transfer with substrate hydroxylation. Recent crystallographic study has also proposed an O_2_‐entry tunnel gated by Pro215 and Trp308 [76]. The HB^A^ protomer in our cryo‐EM structure shows similar rearrangements at these gate residues (Figure S14), although additional studies are needed to establish whether this route is physiologically relevant.

## 3 Conclusion

The cryo‐EM structures presented in this study elucidate the dynamic and asymmetric mechanism underlying sMMO catalysis. Our structures demonstrate that the MMOH–MMOB complex predominantly adopts H‐1B assembly in solution (Figure 1B), which represents a candidate for the catalytically relevant state. A single MMOB molecule binds to one protomer of the MMOH dimer, initiating a cascade of conformational changes that span from the β‐subunit to the di‐iron active site (Figure 2F). The resulting reorganization compresses the Fe···Fe distance to 2.7 Å in the active protomer, consistent with a geometry conducive to O_2_ activation and Q‐intermediate formation (Figure 3A). This rearrangement involves Thr213, which serves as a molecular switch by shifting the local environment from hydrophilic to hydrophobic, thereby generating a continuous substrate‐access pathway in the active protomer (Figure 4). These modifications occur exclusively in the MMOB‐bound protomer, underscoring the functional asymmetry of the MMOH dimer. We further discover that this asymmetry is regulated by the γ‐subunit, which supports the catalytic cycle by stabilizing the compressed active site.

**FIGURE 5:**
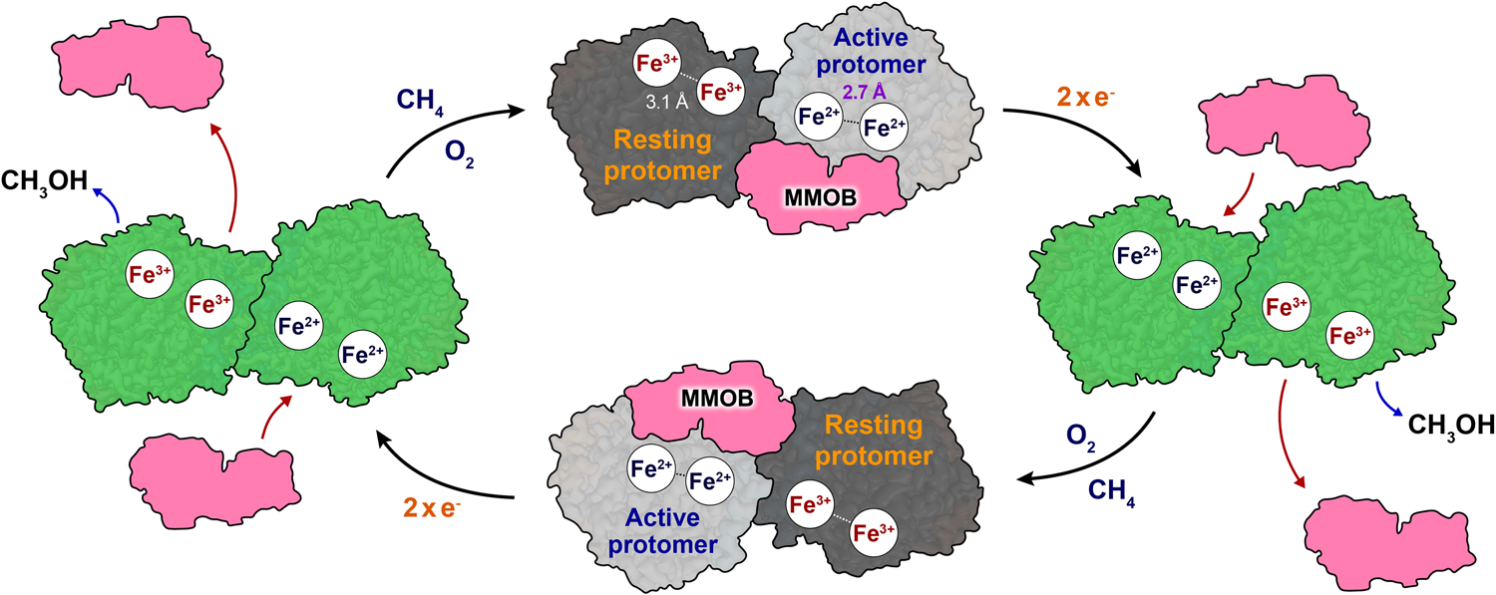
Schematic illustration of the proposed sMMO catalytic cycle driven by sequential MMOB binding. The green structures represent MMOH in its unbound state. Upon MMOB (pink) binding to the canyon region of one protomer, it becomes active (gray), while the other protomer remains in resting state (black). This asymmetric binding pattern supports a coordinated catalytic cycle, enabling continuous methane hydroxylation through alternating MMOB binding and electron transfer events. A conformational shift of Thr213 allows cavities 1 and 2 to connect in the active protomer, while the resting protomer retains a disconnected cavity.

Asymmetry within the H‐1B complex underlies the proposed sMMO mechanism, in which the two protomers alternate between active and resting states (Figure 5). The dynamic interplay between MMOB and the protomers is consistent with a heartbeat‐like manner that could sustain efficient methane hydroxylation. MMOB binds to one protomer and may induce structural modifications that prevent its association with the opposite protomer, thereby enabling MMOR binding. The cryo‐EM structures further support the possibility of simultaneous binding of MMOB and MMOR. Tryptophan quenching experiments demonstrate that the presence of MMOB does not influence the binding affinity of MMOR to MMOH (Figure S15). The binding affinities of MMOR to MMOH are measured as *K*_d1_ = 0.29 ± 0.03 µm and *K*_d2_ = 17.53 ± 0.84 µm. In the presence of MMOB, similar affinities are observed (*K*_d1_ = 0.31 ± 0.04 µm and *K*_d2_ = 16.14 ± 0.65 µm), indicating that MMOB does not interfere with MMOR's interaction with MMOH. These findings provide both structural and functional support for an asymmetric mechanism involving each protomer, with MMOB and MMOR coordinating to regulate the catalytic cycle.

In the proposed catalytic model, MMOR binds to the resting protomer and transfers electrons to Fe^3+^−Fe^3+^ active site, which leads to a transition to Fe^2+^−Fe^2+^ (Figure 5). MMOR dissociates from the MMOH canyon region following electron transfer, after which MMOB binds to convert the resting protomer into its active form. The active protomer then performs methane hydroxylation and releases both MMOB and the product, methanol. This asymmetric nature allows the protomers to alternate roles and maintain continuous hydroxylation. The cryo‐EM structures demonstrate the conformational asymmetry and dynamic interaction required to sustain this catalytic mechanism. These findings significantly advance the understanding of the mechanism of sMMO, establishing a new foundation for further studies on enzymatic regulation and methane hydroxylation.

## 4 Experimental Section

### 4.1 Fermentation and Purification of MMOH

MMOH from *M. sporium* 5 was purified as previously described [14, 19, 39]. MMOH was expressed through *M. sporium* 5 in a copper‐limited nitrate minimal salt (NMS) medium at 37°C in a 5 L incubator to achieve an optical density at 600 nm (OD_600_) of 6–8 under a methane:air (v/v) ratio of 1:4. The pH was adjusted to 7.0 using 100 mm NaOH or HCl. Cells were harvested by centrifugation at 11 355 × g for 20 min at 4°C. The cell pellets were resuspended in buffer A (25 mm MOPS, 25 mm NaCl, 5 mm MgCl_2_, 2 mm L‐cysteine, 8 mm sodium thioglycolate, 200 µm (NH_4_)_2_Fe(SO_4_)_2_·6H_2_O, 0.25 µL/mL DNase I, and 0.04 mg/mL PMSF; pH 6.5) and sonicated for 40 min (pulse cycle: 15 s on and 45 s off). The filtered supernatant was subjected to sequential purification using DEAE‐Sepharose Fast Flow, Superdex 200, and Q‐Sepharose Fast Flow (Cytiva) columns on an ÄKTA Pure 25 L fast protein liquid chromatography system (Cytiva) to purify MMOH.

### 4.2 Expression and Purification of MMOB

MMOB from *M. sporium* 5 was purified as previously described [14, 39, 77]. To express MMOB, the *pET30a(+)‐mmoB* plasmid was transformed into BL21(DE3) cells. Transformants were grown overnight at 37°C with shaking (200 rpm), then further incubated in 500 mL LB medium containing 50 µg/mL kanamycin. At an OD_600_ of 0.6–0.7, MMOB expression was induced using 0.1 mm IPTG at 37°C with shaking (200 rpm) for 4 h. The induced cell cultures were harvested by centrifugation at 11 355 × g for 20 min at 4°C. The resulting pellet (from 1 L culture) was resuspended in buffer B (25 mm sodium phosphate, 75 mm NaCl, 5 mm MgCl_2_, 1 mm Na_2_‐EDTA, 1 mm DTT, 0.25 µL/mL DNase I, and 0.04 mg/mL PMSF; pH 6.0) and sonicated for 40 min (pulse cycle: 15 s on and 45 s off). The filtered supernatant was purified using Q‐Sepharose Fast Flow and Superdex 75 (Cytiva) columns on an ÄKTA Pure 25 L fast protein liquid chromatography system (Cytiva) to obtain MMOB.

### 4.3 Expression and Purification of MMOR

MMOR from *M. sporium* 5 was purified as previously described [14, 39, 77]. To express MMOR, the *pET30a(+)‐mmoC* plasmid was transformed into Rosetta(DE3) cells. Transformants were grown overnight at 37°C with shaking (200 rpm), then further incubated in 500 mL LB medium containing 50 µg/mL kanamycin. At an OD_600_ of 0.6–0.7, MMOR expression was induced using 0.1 mM IPTG at 25°C with shaking (200 rpm) for 6 h. The induced cell cultures were harvested by centrifugation at 11 355 × g for 20 min at 4°C. The resulting pellet (from 2 L culture) was resuspended in buffer C (25 mm MOPS, 50 mm NaCl, 5 mm MgCl_2_, 2 mm L‐cysteine, 8 mm sodium thioglycolate, 200 µm (NH_4_)_2_Fe(SO_4_)_2_·6H_2_O, 0.25 µL/mL DNase I, and 0.04 mg/mL PMSF; pH 6.5) and sonicated for 40 min (pulse cycle: 15 s on and 45 s off). The filtered supernatant was purified using Q‐Sepharose Fast Flow and Superdex 75 (Cytiva) columns connected to an ÄKTA Pure 25 L fast protein liquid chromatography system (Cytiva) to obtain MMOR.

### 4.4 Cryo‐EM Grid Preparation and Data Collection

The sMMO components—including MMOH, MMOB, and MMOR—purified from *M. sporium* 5 exhibited a specific enzyme activity of 400–500 mU·mg^−1^ using propylene as a substrate, with NADH consumption showing substrate dependence and only minimal NADH oxidation observed in the absence of hydrocarbon substrate [14, 39, 45]. Cryo‐EM grids were prepared at the Institute for Basic Science (IBS, South Korea) and the Institute for Bioscience and Biotechnology Research (IBBR, USA). Purified MMOH and MMOB were stored in buffer D (25 mm MOPS, 50 mm NaCl, and 1 mm DTT; pH 6.5). The MMOH–MMOB (H‐B) complex was prepared at a 1:2.2 molar ratio. MMOH or H‐B was diluted to 2 mg/mL and applied onto glow‐discharged holey carbon grids (Quantifoil; Cu 200 mesh, R 1.2/1.3; negative discharge for MMOH, positive for H‐B). After blotting with filter paper, the grids were vitrified in liquid ethane using a Vitrobot Mark IV (Thermo Fisher Scientific; TFS, USA) at 4°C and 100% relative humidity. Cryo‐EM images were collected using a 300 kV Krios G4 (TFS, USA) equipped with a BioQuantum K3 detector (Gatan Inc, USA) and EPU software (TFS, USA). Further details are provided in Table S1.

### 4.5 Cryo‐EM Data Processing of MMOH Alone

All datasets were processed using cryoSPARC (v.4.2.1) [26]. The collected movies were divided into two datasets and pre‐processed using patch motion correction and patch contrast transfer function (CTF) estimation. Blob picking from the first dataset yielded 4,779,030 particles, which were extracted using a 320‐pixel box [25]. After two rounds of 2D classification, ab‐initio reconstruction generated two classes corresponding to the putative αβγ protomer and α_2_β_2_γ_2_ homodimer. Particles corresponding to the homodimer were refined through 3D classification, and 236,211 particles were selected for final reconstruction. The resulting particles were re‐extracted into 320‐pixel boxes. An identical processing workflow was applied to the second dataset, and an additional 171,748 particles were acquired. The selected particles from the two datasets were combined and reconstructed with C_2_ symmetry, producing a 3D electron density map at 2.64 Å, where the resolution was estimated via Fourier shell correlation at the 0.143 criterion. The structural dynamics of MMOH were evaluated through 3D flexible refinement using the final particles [32].

### 4.6 Cryo‐EM Data Processing of MMOH–1MMOB Complex

Data processing was performed using cryoSPARC (v.3.3.1) [26]. All movies were pre‐processed using Patch motion correction and Patch CTF estimation. Blob picking resulted in 7,714,850 particles, which were extracted using a 360‐pixel box [25]. After four rounds of 2D classification, over‐sampled views were removed, which resulted in the selection of 1,535,094 particles [78]. The resulting particles were re‐extracted into 320‐pixel boxes. After one round of 2D classification, high‐quality particles were selected and refined using global and local CTF refinement. The resulting particles were subjected to 3D variability analysis (3DVA) and 3D classification, respectively [31]. The 3DVA was performed using 650,028 particles, which resulted from the symmetry expansion of 325,014 particles around the C_2_ symmetry, with a low‐pass filter resolution of 3 Å. A 3D variability display was conducted to generate volumes and particles from each of the 20 clusters that fit the reaction coordinates.

### 4.7 Model Building and Refinement

MMOH_ox_ (PDB: 1MTY) and X‐ray MMOH–2MMOB (PDB: 4GAM) structures were fitted into EMD‐39540 and EMD‐38391, respectively, using UCSF Chimera for modeling of MMOH and the MMOH‐1MMOB complex [17, 72, 79]. The model was refined through multiple cycles of real‐space refinement in Phenix using geometric and secondary structure restraints, followed by manual adjustments in Coot [80, 81, 82]. Overall model validation was performed using the comprehensive validation tool in Phenix [83]. Statistics for cryo‐EM data collection, 3D reconstruction, and model refinement are summarized in Table S1. Structural figures were generated using PyMOL (v.2.5.2) and UCSF Chimera [82, 84].

### 4.8 Measurements of Binding Affinities of MMOH with MMOR under MMOB Conditions

Binding affinities between MMOH and MMOR were assessed via tryptophan fluorescence quenching using a spectrofluorometer (JASCO, FP‐8300), with excitation at 282 nm and emission at 336 nm. One equivalent of MMOH (2.56×10^−10^ mol) in buffer D was titrated with MMOR to saturation, both in the presence and absence of one equivalent of MMOB. The 1:2 binding model was fitted to measure the binding affinities between MMOH and MMOR as previously described. Curve fitting was performed using Origin2018 (OriginLab) and Mathematica (Wolfram).

## Author Contributions

Y.H., H.J.H. and J.‐G.N. fermented *M. sporium* 5 and expressed protein; Y.H., S.P., C.G.S., and H.G.K. purified the protein; Y.H., B.R., E.P., and S.J.L. performed electron microscopy; Y.H., B.R., C.G.S., E.P., and S.J.L. processed the electron microscopy images and built the model; Y.H., S.P., D.L., and S.J.L. analyzed the coordination studies; Y.H., S.P., D.L., and S.J.L. measured the binding affinities between MMOH and MMOR; E.P. and S.J.L. designed the experiments, compiled the manuscript, and edited and reviewed the manuscript with input from all the authors.

## Funding

NRF‐2015M3D3A1A01064876, 2025‐RISE‐13‐JBU, RS‐2024‐00440289, RS‐2025‐23292974.

## Conflicts of Interest

The authors declare no conflicts of interest.

## Supporting information




**Supporting File 1**: advs73942‐sup‐0001‐SuppMat.docx.

**Supporting File 2**: advs73942‐sup‐0002‐VideoS1.mp4.

**Supporting File 3**: advs73942‐sup‐0003‐VideoS2.mp4.

**Supporting File 4**: advs73942‐sup‐0004‐VideoS3.mp4.

**Supporting File 5**: advs73942‐sup‐0005‐VideoS4.mp4.

**Supporting File 6**: advs73942‐sup‐0006‐VideoS5.mp4.

**Supporting File 7**: advs73942‐sup‐0007‐VideoS6.mp4.

**Supporting File 8**: advs73942‐sup‐0008‐VideoS7.mp4.

**Supporting File 9**: advs73942‐sup‐0009‐VideoS8.mp4.

## Data Availability

The data supporting this study are available from the corresponding authors upon request. The cryo‐EM structures and the atomic models of the MMOH and MMOH–1MMOB complexes have been deposited in the Electron Microscopy Data Bank (EMDB) and Protein Data Bank (PDB), respectively. The accession codes are as follows: EMD‐39540 and PDB ID 8YRD for the cryo‐EM structure of MMOH from *M. sporium* 5; and EMD‐38391 and PDB ID 8XIW for the cryo‐EM structure of MMOH–1MMOB complex from *M. sporium* 5.
