# Supplementary material for: Cryo‐EM Structures Reveal the Molecular Basis of Asymmetric Allosteric Activation by MMOB in the Hydroxylase of Soluble Methane Monooxygenase
Source: Adv Sci (Weinh). 2026 Jan 21;13(19):e17312. doi: 10.1002/advs.202517312 (PMC13045389; doi:10.1002/advs.202517312)
Supplement: Supplementary file 1 — Supporting File 1: advs73942‐sup‐0001‐SuppMat.docx. [file ADVS-13-e17312-s002.docx]

Supporting Information

for

**Cryo-EM Structures Reveal the Molecular Basis of Asymmetric Allosteric Activation
by MMOB in the Hydroxylase of Soluble Methane Monooxygenase**

*Yunha Hwang**^+[a]^, Bumhan Ryu^+[b]^, Soyeon Park^[a]^, Dong-Heon Lee^[a]^, Hyo Jin Hong^[c]^, Jeong-Geol Na^[c]^, Chul Gyu Song^[d]^, Hyun Goo Kang^[e]^, Edwin Pozharski^*[f.g]^, and Seung Jae Lee**^*[a,h]^*

^a^Department of Chemistry, Jeonbuk National University, Jeonju 54896, Republic of Korea

^b^Research Solution Center, Institute for Basic Science, Daejeon 34126, Republic of Korea

^c^Department of Chemical and Biomolecular Engineering, Sogang University, Seoul 04107, Republic of Korea

^d^Institute for ICT-Based Infectious Disease Technology Research and Department of Electronic Engineering, Jeonju 54896, Republic of Korea

^e^Department of Neurology and Research Institute of Clinical Medicine, Jeonbuk National University, Jeonju 54896, Republic of Korea

^f^Department of Biochemistry and Molecular Biology, School of Medicine, University of Maryland, Baltimore, MD 21201, United States of America

^g^Institute for Bioscience and Biotechnology Research, University of Maryland, Rockville, MD 20850, United States of America

^h^Research Institute of Molecular Biology and Genetics, Jeonbuk National University, Jeonju 54896, Republic of Korea

*To whom correspondence should be addressed: epozharskiy@som.umaryland.edu and slee026@jbnu.ac.kr

^+^These authors contributed equally to this work

**Table of Contents**

**Figure S1** Flow chart for cryo-electron microscopy (cryo-EM) image processing

for the MMOH–MMOB complex S3

**Figure S2** H-2B complex derived from stacked particles of *Methylosinus sporium* 5 S4

**Figure S3** High flexibility like a “beating heart” of MMOH in native state with cryo-EM S5

**Figure S4** Helix nomenclature in the α- and γ-subunits of MMOH with residue ranges S6

**Figure S5** Comparison of conformational changes in the MMOH α-subunit

upon MMOB binding S7

**Figure S6** Helices that undergo significant conformational shifts upon MMOB binding S8

**Figure S7** MMOB induced conformational changes in the four-helix bundles

of the MMOH α-subunit from cryo-EM S10

**Figure S8** Conformational changes in MMOH induced by MMOB binding S11

**Figure S9** MMOH γ-subunit-mediated stabilization of conformational changes

induced by MMOB binding S12

**Figure S10** Di-iron active site in MMOH from X-ray crystallography S13

**Figure S11** Di-iron active site in sMMO complex from X-ray crystallography

and X-ray free electron laser S14

**Figure S12** The key residues of the MMOH α-subunit related to the di-iron active site

upon MMOB binding with cryo-electron microscopy S15

**Figure S13** Analysis of cavities in cryo-EM structures of sMMO S16

**Figure S14** Conformational rearrangements of the proposed O_2_-entry tunnel gate residues

in the cryo-EM H-1B structure. S17

**Figure S15** Titration of MMOR to MMOH in the presence and absence of MMOB S18

**Table S1** Cryo-EM data collection, refinement, and validation statistics S19

**Table S2** Metal coordination in the active sites of different forms of MMOH S20

**Video S1-3** Structural change of MMOH induced by MMOB with 3D variability analysis S21

**Video S4-6** Monitoring the motion of MMOH through 3D flexible refinement S21

**Video S7** MMOB–induced structural dynamics of the di-iron active site revealed

by 3D variability analysis S21

**Video S8** MMOB–induced structural dynamics of residues involved in cavities 1–2

connectivity revealed by 3D variability analysis. S21

**References** S22


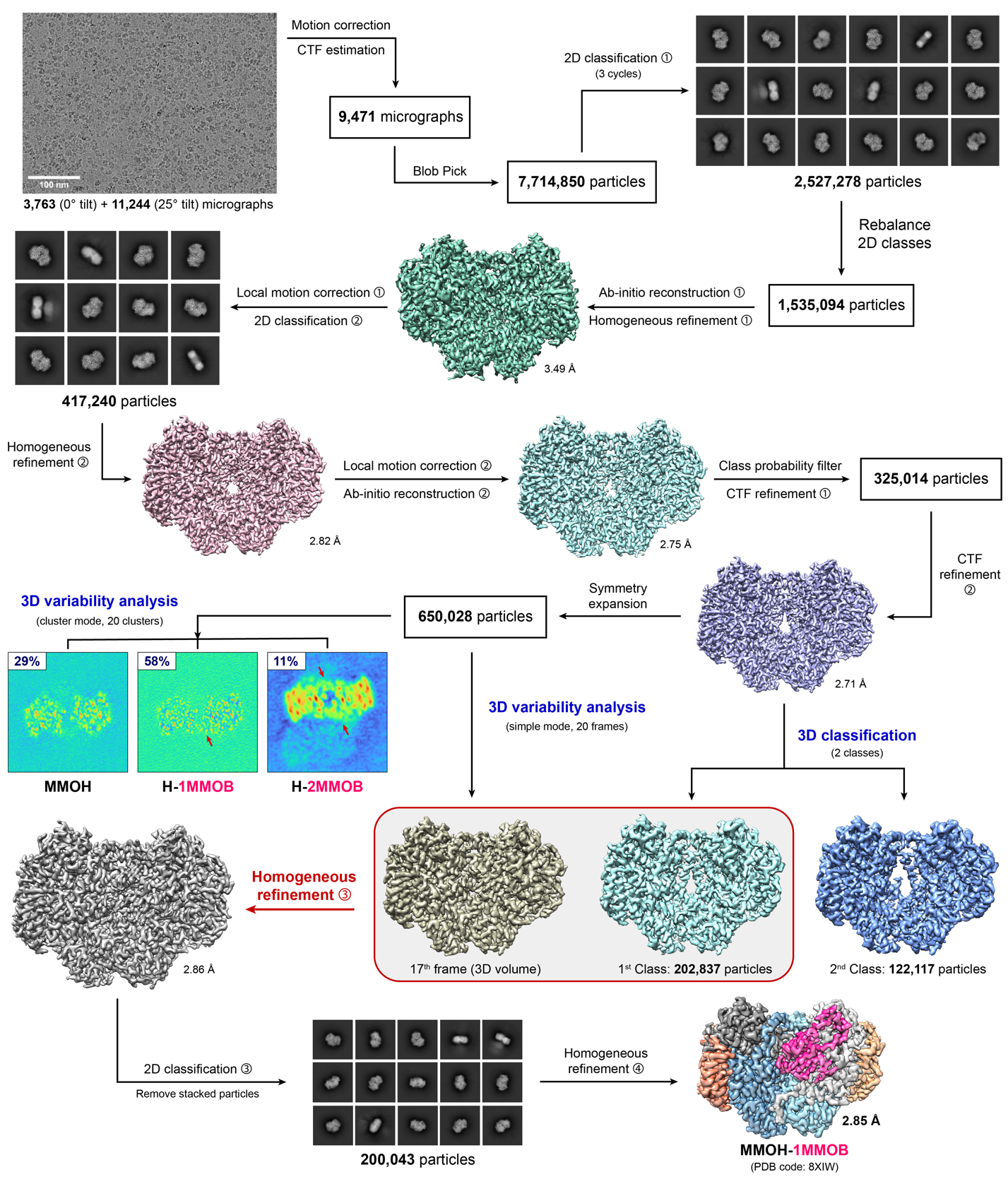


**Figure S1.** Overall flow chart for cryo-electron microscopy (cryo-EM) image processing of the MMOH–MMOB complex (H-1B, PDB: 8XIW). This workflow presents the complete data-processing pipeline corresponding to the simplified summary shown in Figure 1A. Red arrows indicate MMOB in 3D variability analysis using cluster mode.^[1]^ The number below the 3D map volume indicates the resolution (unit: Å). In the native state, the H-B complex indicated that only one MMOB bound to MMOH.

**
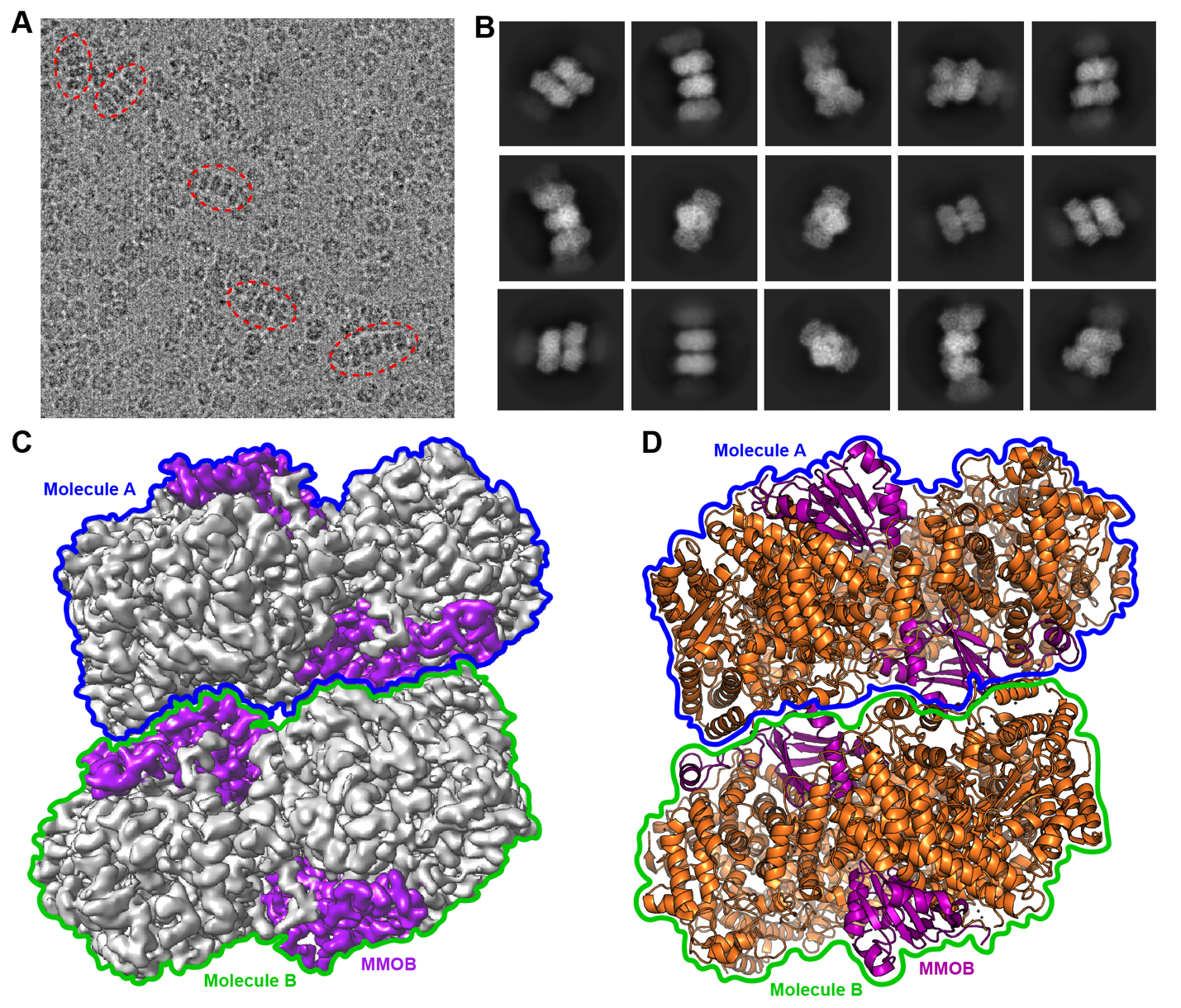
**

**Figure S2.** H-2B complex derived from stacked particles of *Methylosinus sporium* 5. (A) Stacked particles of the H-2B complex in a micrograph. The H-2B complexes stacked in a row are indicated by red dashed circles. (B) The 2D classes of stacked particles of the H-2B complex. (C and D) Comparison of (C) the 3D cryo-EM volume of the stacked H-2B complex and (D) the X-ray structure (PDB: 4GAM).^[2]^ MMOB is depicted in purple, while MMOH is depicted in gray (cryo-EM) and orange (X-ray crystallography). Each stacked MMOH–MMOB complex consisted of two molecules (molecule A, blue; molecule B, green), with each MMOH bound to two MMOB.

**
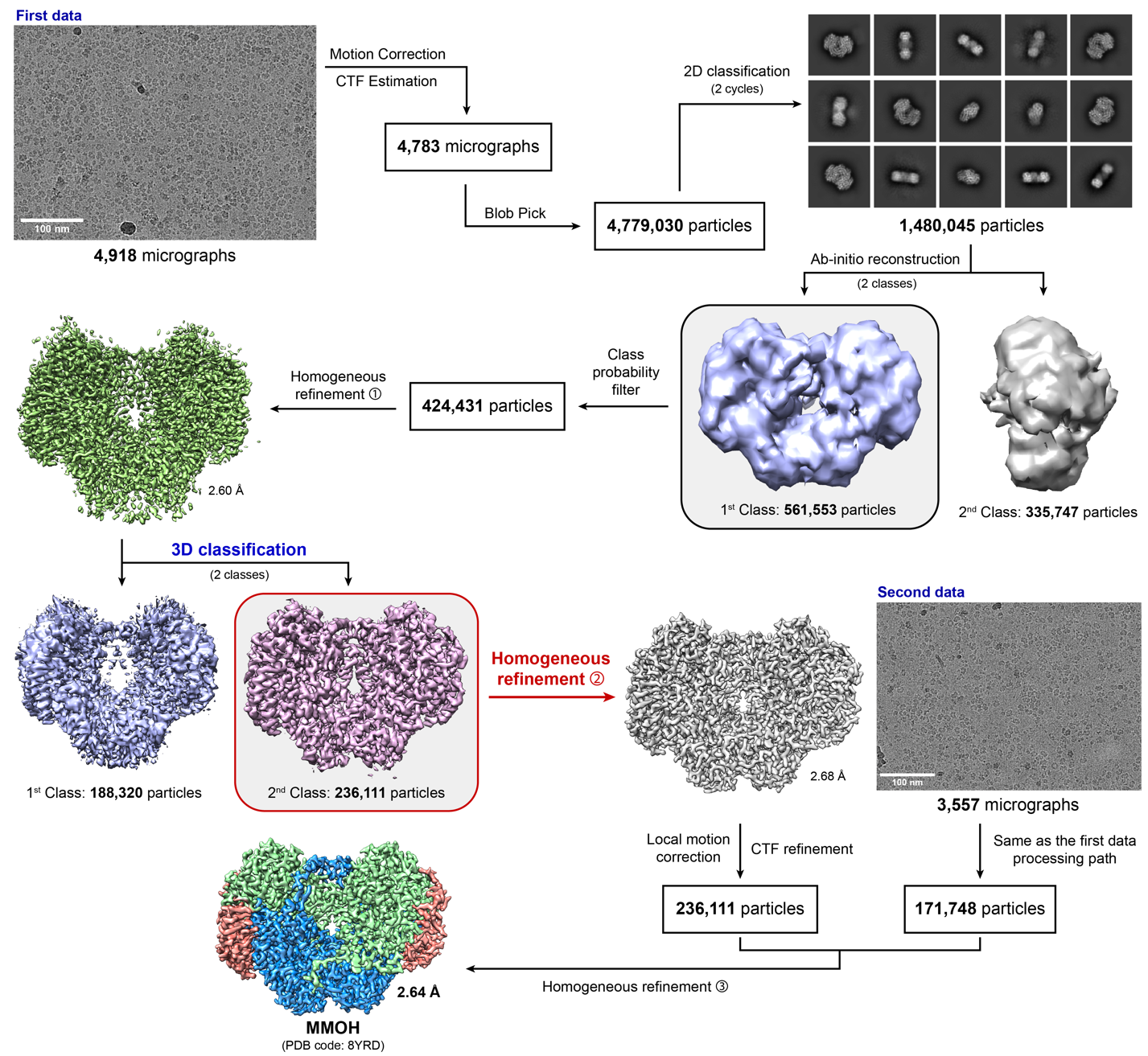
**

**Figure S3.** High flexibility like a “beating heart” of MMOH in native state with cryo-EM. Flow chart for cryo-EM image processing for MMOH (PDB: 8YRD). The numbers next to the 3D map volumes indicate the resolution (unit: Å). As a result of data processing, the 3D map volume of MMOH was obtained with a 2.64 Å resolution.

**
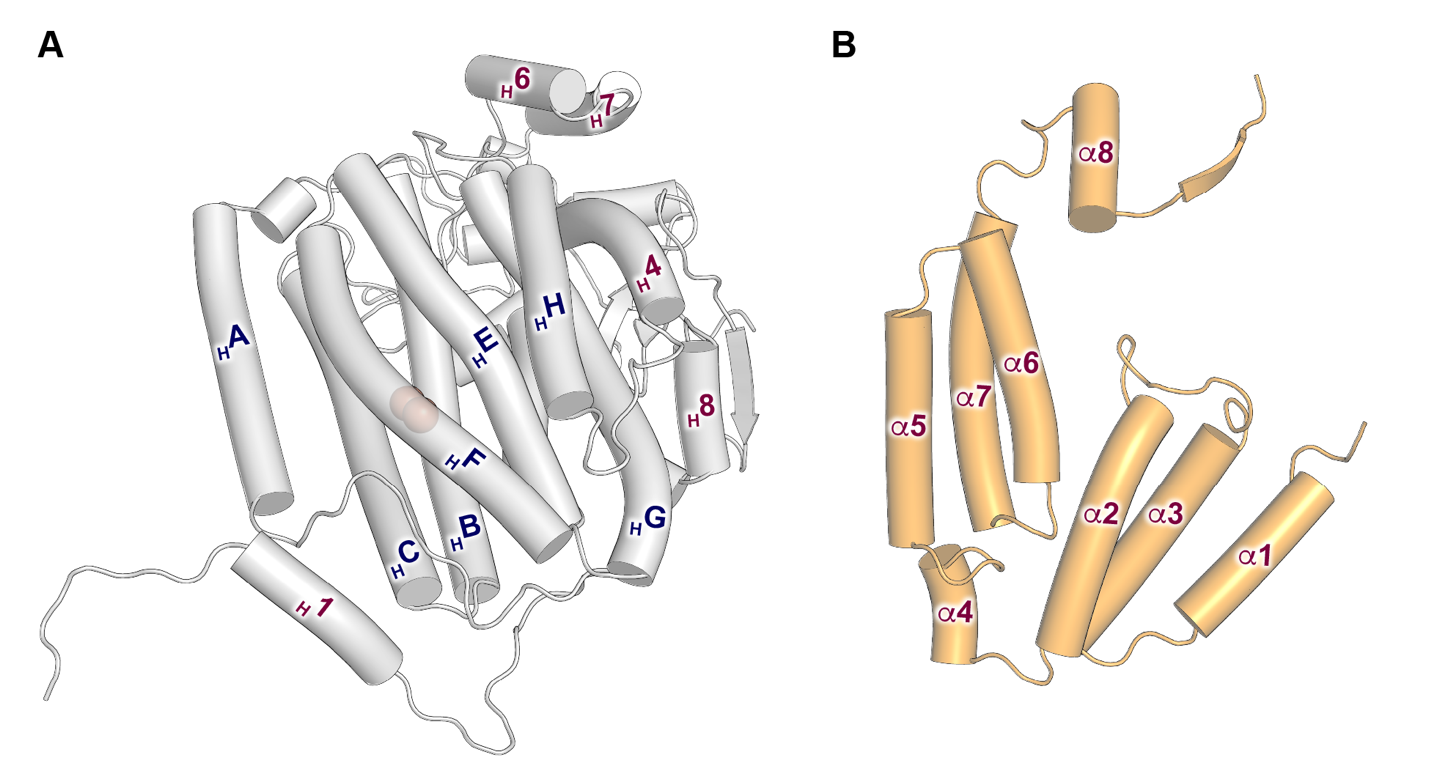
**

**Figure S4.** Helix nomenclature in the α- and γ-subunits of MMOH with residue ranges. (A) Helices of the MMOH α-subunit are labeled according to the convention established in the first X-ray structure of MMOH^[3]^, with long helices designated by letters (A–H) and shorter helices by numbers: Helix A (64–89), Helix B (97–127), Helix C (131–160), Helix D (179–193), Helix E (197–227), Helix F (230–257), Helix G (261–292), Helix H (301–321), Helix 4 (332–352), and Helix 8 (450–460). The subscript “H” before an English letter indicates a helix. (B) Helices of the MMOH γ-subunit are numbered with an α-prefix to distinguish them from the α-subunit helices: α1 (11–22), α2 (26–39), α3 (54–71), α4 (75–80), α5 (87–101), α6 (105–121), α7 (126–145), and α8 (153–160).

**
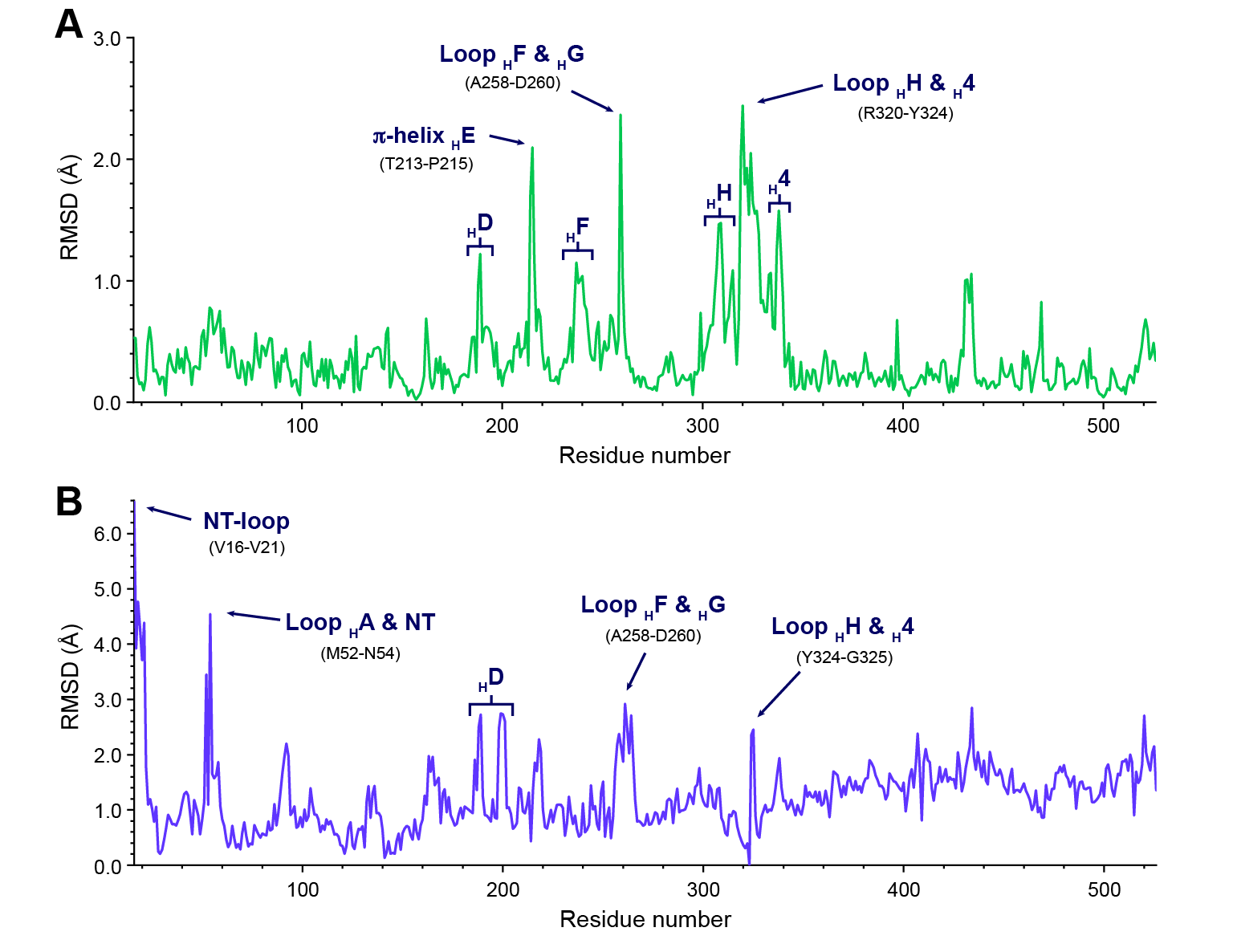
**

**Figure S5.** Comparison of conformational changes in the MMOH α-subunit upon MMOB binding. (A-B) Root-mean-square deviation (RMSD) of the main chain (C_α_) per residue for the cryo-EM HB^A^ with (A) cryo-EM HB^B^ and (B) the X-ray H-2B complex (PDB: 4GAM).^[2]^ Graph of RMSD per residue plotted using visual molecular dynamics (VMD, v.1.9.4a57) and the MultiSeq program.^[4]^


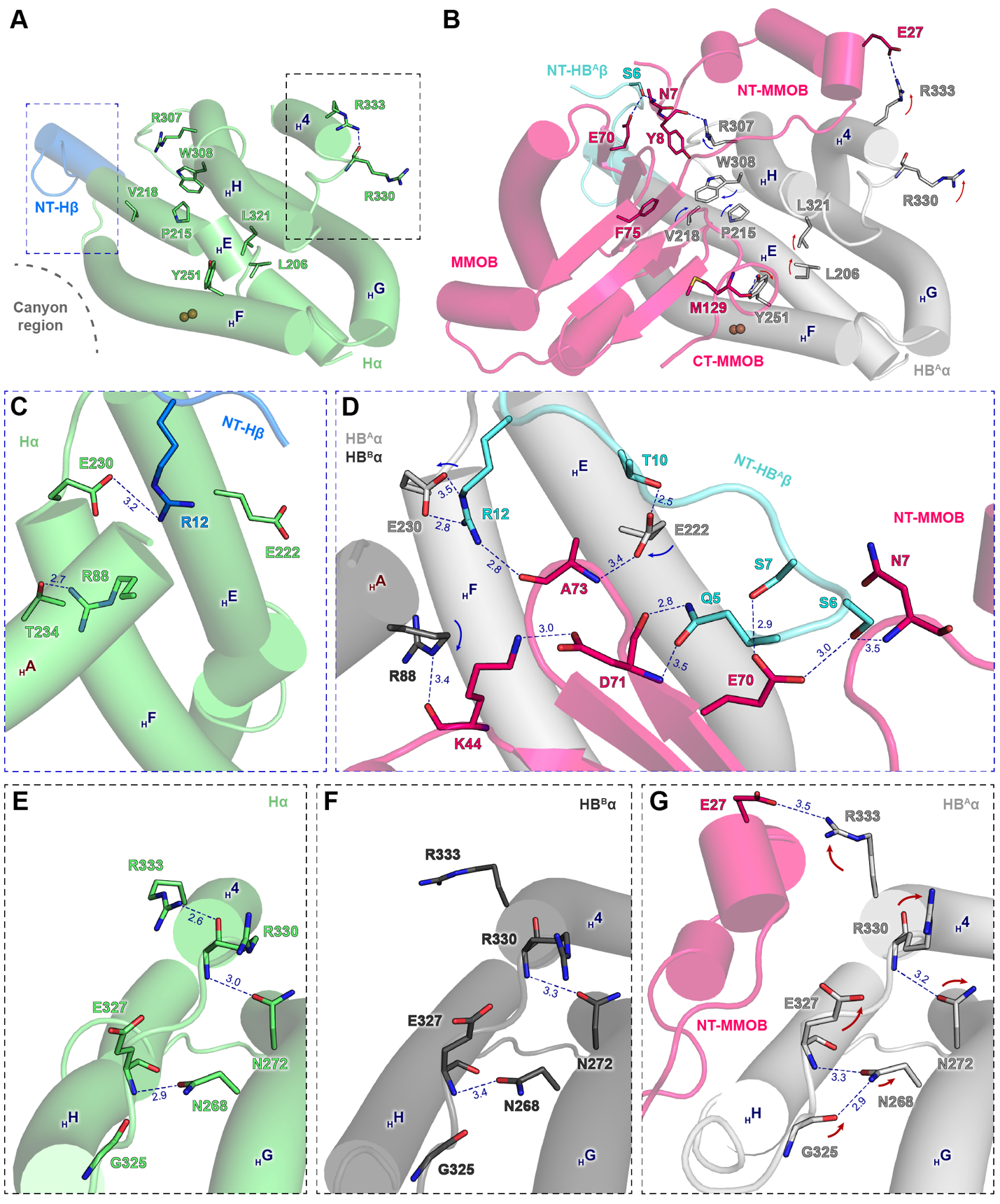


**Figure S6.** Helices that undergo significant conformational shifts upon MMOB binding. (A) cryo-EM MMOH (PDB: 8YRD). (B) cryo-EM HB^A^ (PDB: 8XIW). The *N*-terminus (NT) of the MMOHβ provides structural support, while both the *C*-terminus (CT) and NT-MMOB contribute to the elevation of the MMOHα. (C and D) Detailed molecular interactions at the canyon region mediated by the NT-MMOHβ in (C) cryo-EM MMOH and (D) cryo-EM H-1B. NT-HB^A^β induces conformational changes in NT-MMOB, helices E and F of protomer A, and helix A of protomer B. (E-G) Detailed molecular interactions at the NT-MMOB with the MMOHα from (E) cryo-EM MMOH, (F) cryo-EM HB^B^, and (G) cryo-EM HB^A^. The red arrows indicate conformational changes caused by NT-MMOB. The color of the arrows indicates conformational changes caused by NT- and CT-MMOB (red) and NT-HB^A^β (blue).

**
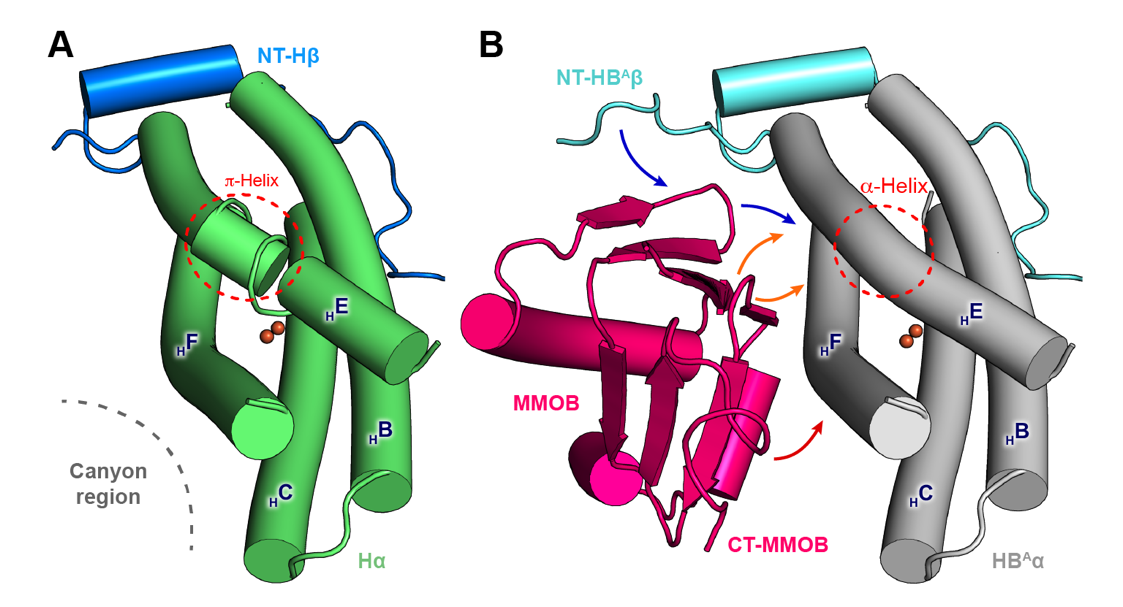
**

**Figure S7.** MMOB induced conformational changes in the four-helix bundles of the MMOH α-subunit from cryo-EM. (A) MMOH. (B) Cryo-EM HB^A^. NT-MMOHβ provides structural support for helices B and C.^[2, 5]^ Due to the interaction of MMOB and NT-HB^A^β with the MMOH canyon region, the π-helix of helix E is converted into an α-helix. The color of the arrows indicates conformational changes caused by CT-MMOB (red), NT-HB^A^β (blue), and the core region of MMOB (orange).

**
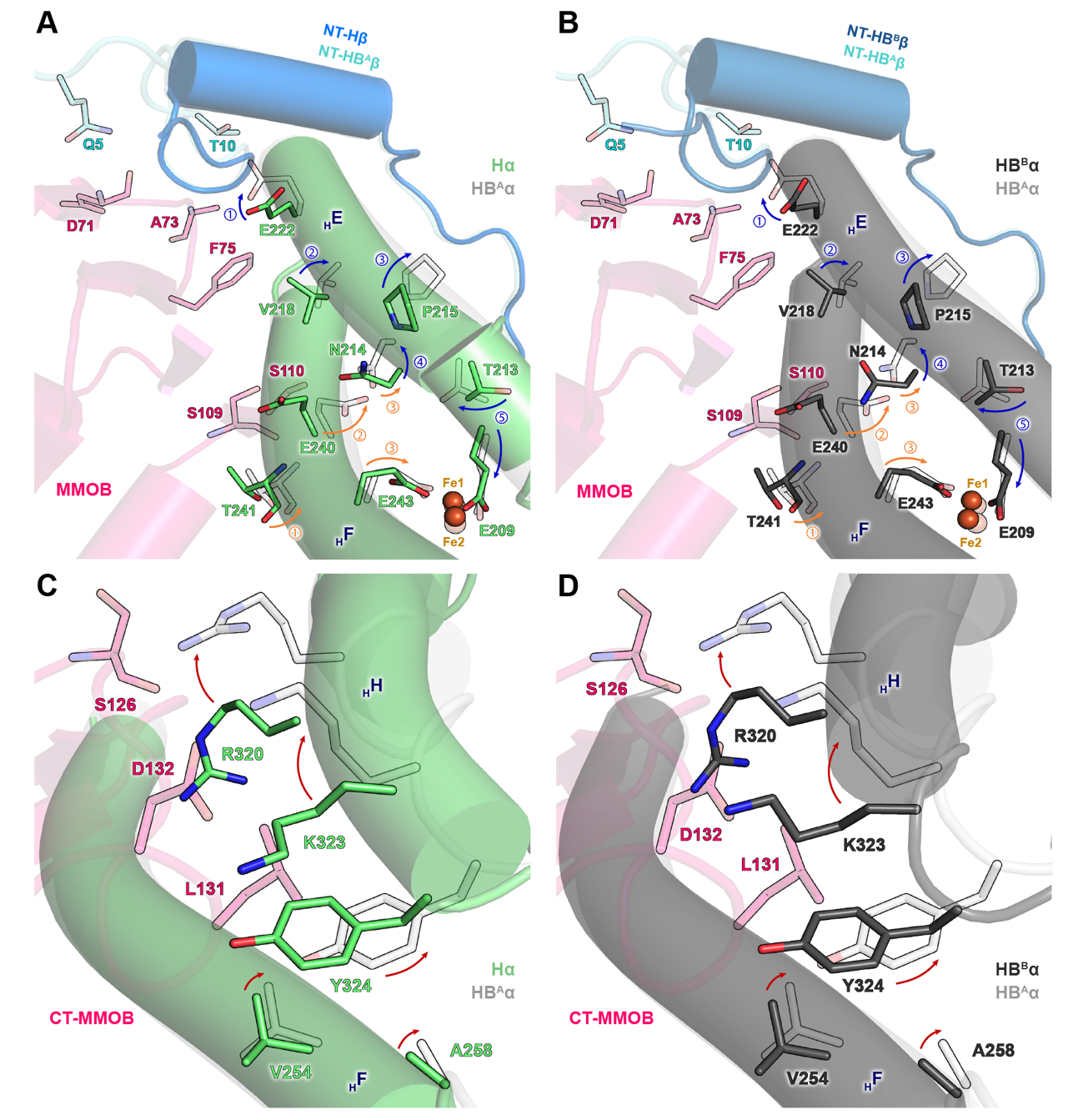
**

**Figure S8.** Conformational changes in MMOH induced by MMOB binding. (A and B) Structural alignment of cryo-electron microscopy (EM) HB^A^ with (A) cryo-EM MMOH and (B) cryo-EM HB^B^ in the canyon region.^[2]^ (C and D) Structural alignment of cryo-EM HB^A^ with (C) cryo-EM MMOH and (D) cryo-EM HB^B^ in C-terminal (CT)-MMOB with helices H and 4. CT-MMOB induces elevation of the MMOH α-subunit. The color of the arrows indicates conformational changes caused by CT-MMOB (red), NT-HB^A^β (blue), and the core region of MMOB (orange).

**
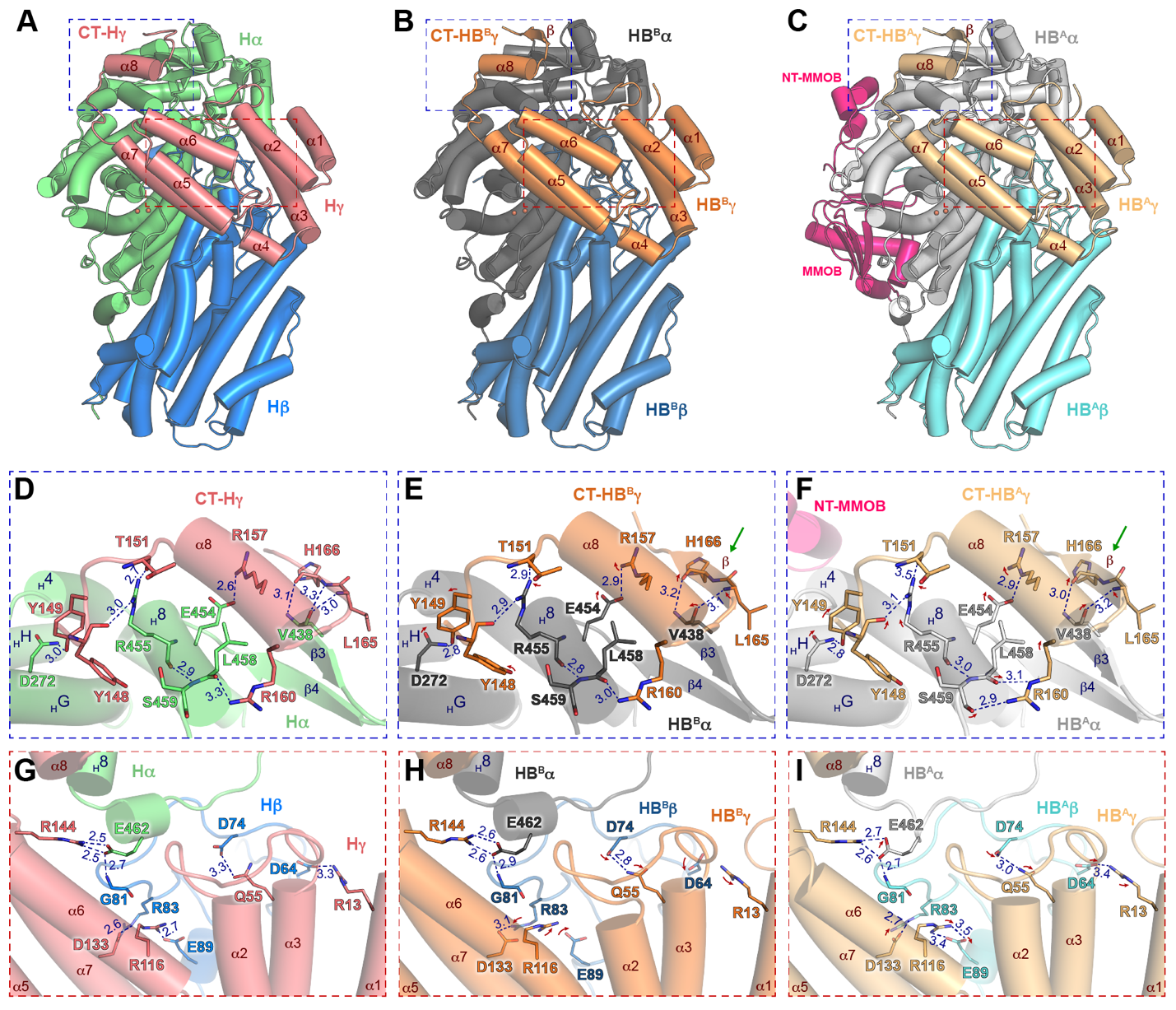
**

**Figure S9.** MMOH γ-subunit-mediated stabilization of conformational changes induced by MMOB binding. (A-C) Overall structures of (A) cryo-EM MMOH (PDB: 8YRD), (B) cryo-EM HB^B^ (PDB: 8XIW), and (C) HB^A^ (PDB: 8XIW). The MMOH γ-subunit (MMOHγ) interacts with both the MMOH α-subunit (MMOHα) and MMOH β-subunit (MMOHβ) to stabilize the overall structure. (D-F) The *C*-terminal region of MMOHγ interacts with MMOHα in (D) MMOH, (E) HB^B^, and (F) HB^A^. (G-I) The core region of MMOHγ interacts with MMOHβ in (G) cryo-EM MMOH, (H) HB^B^, and (I) HB^A^. Red arrows exhibit conformational changes caused by MMOB and green arrows indicate β-strand secondary structure of MMOHγ formed by MMOB binding.

**
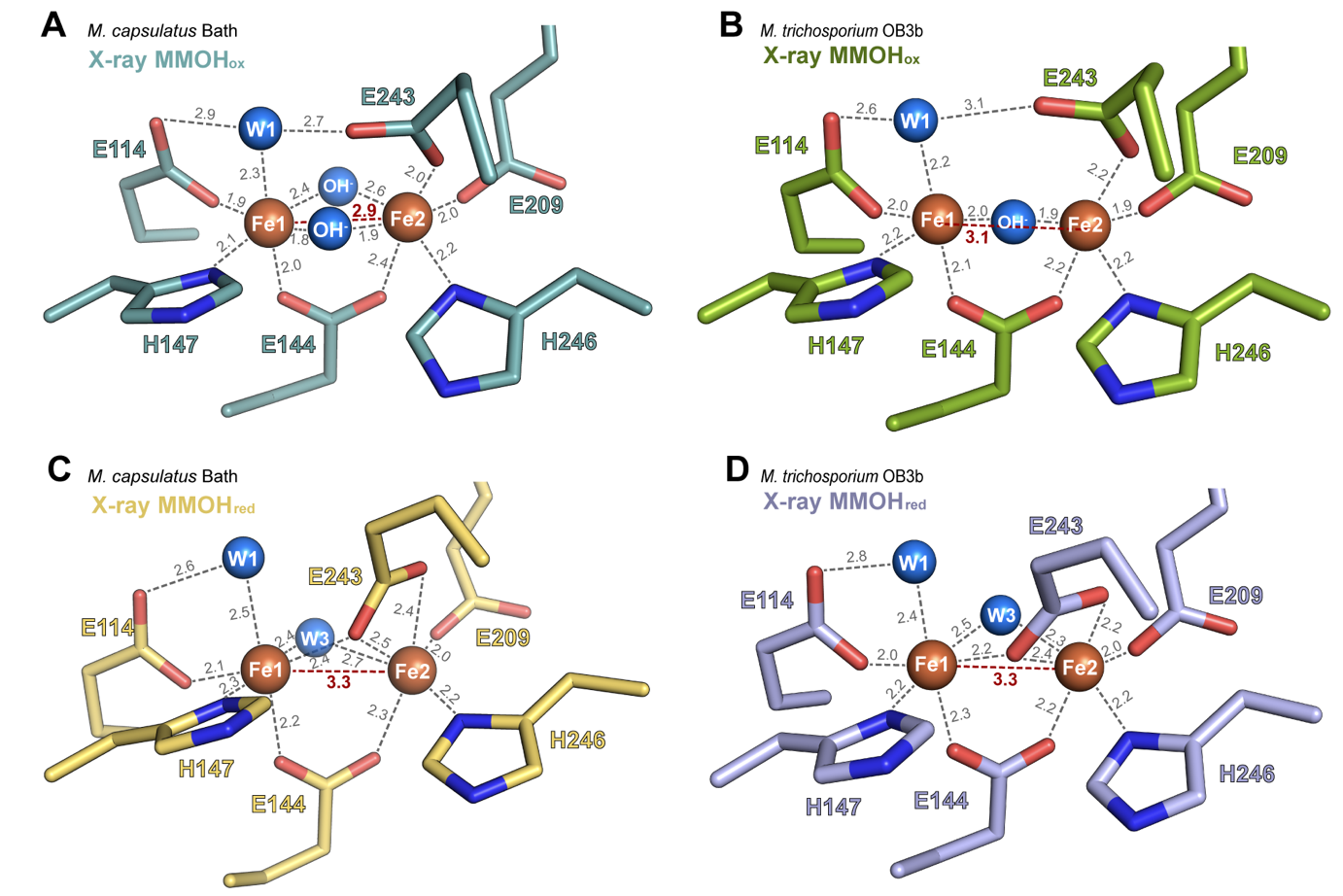
**

**Figure S10.** Di-iron active site in MMOH from X-ray crystallography. (A) X-ray, oxidized MMOH from *Methylococcus capsulatus* Bath (PDB: 1MTY, teal).^[6]^ (B) X-ray, oxidized MMOH from *Methylosinus trichosporium* OB3b (PDB: 6VK6, splitpea).^[7]^ (C) X-ray, reduced MMOH from *Methylococcus capsulatus* Bath (PDB: 1FYZ, yellow-orange).^[8]^ (D) X-ray, reduced MMOH from *Methylosinus trichosporium* OB3b (PDB: 6VK7, lightblue).^[7]^ The two Fe atoms are coordinated by six residues, including four glutamates and two histidines. Water molecules are displayed as marine spheres and are numbered according to their positions. The coordination distances are depicted in gray, and the Fe–Fe distances are represented in red. The unit of distance is Ångström (Å).

**
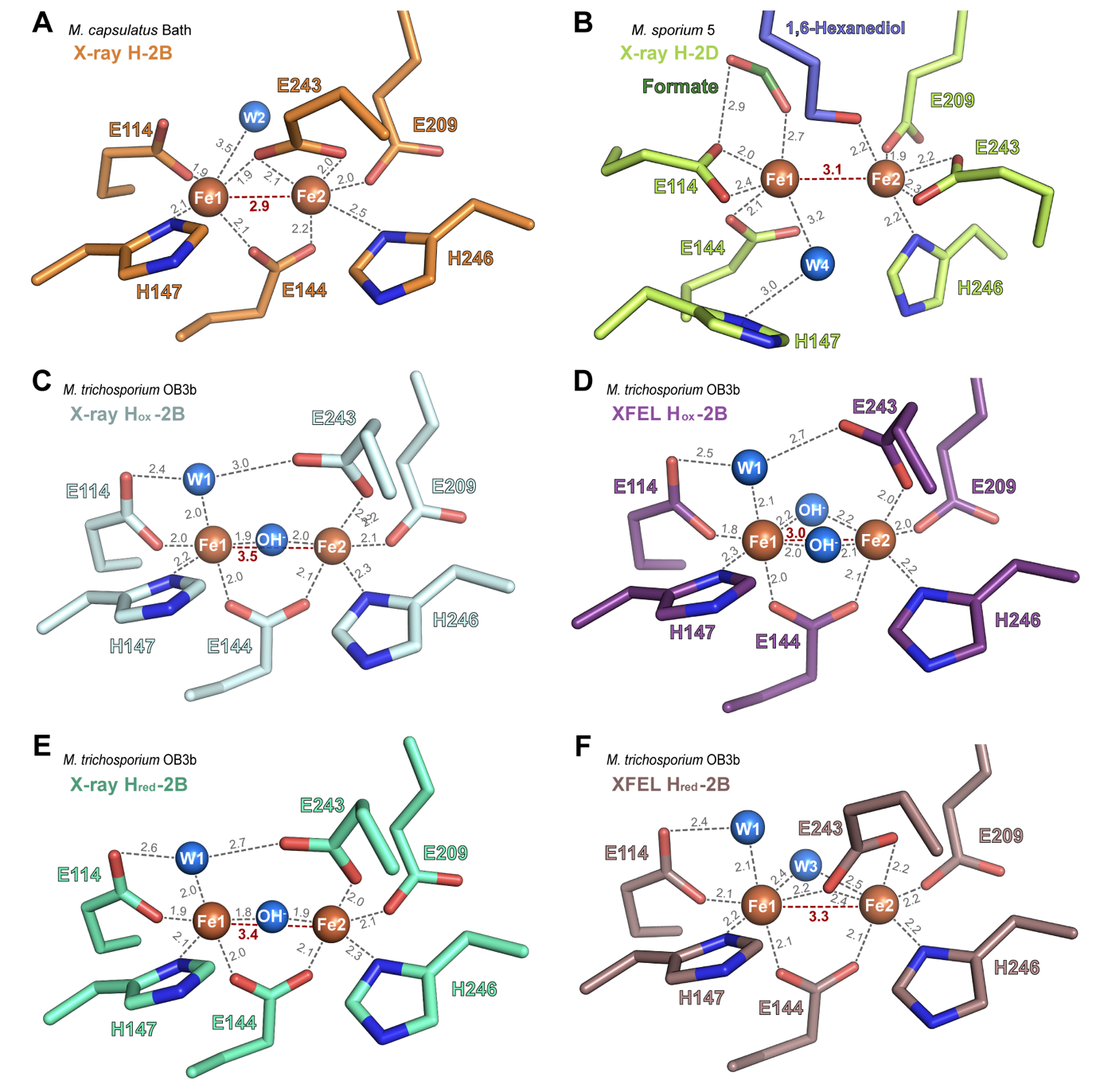
**

**Figure S11.** Di-iron active site in sMMO complex from X-ray crystallography and X-ray free electron laser (XFEL). (A) X-ray, MMOH-2MMOB complex from *Methylococcus capsulatus* Bath (PDB: 4GAM, tv_orange).^[2]^ (B) X-ray, MMOH-2MMOD complex from *Methylosinus sporium* 5 (PDB: 6D7K, lemon).^[5]^

(C) X-ray, oxidized MMOH-2MMOB complex from *Methylosinus trichosporium* OB3b (PDB:6VK5, palecyan).^[7]^ (D) XFEL, oxidized MMOH-2MMOB complex from *Methylosinus trichosporium* OB3b (PDB: 6YD0, resolution: 1.95 Å, violet-purple).^[9]^ (E) X-ray, reduced MMOH-2MMOB complex from *Methylosinus trichosporium* OB3b (PDB: 6VK4, greencyan).^[7]^ (F) XFEL, reduced MMOH-2MMOB complex from *Methylosinus trichosporium* OB3b (PDB: 6YDI, resolution: 1.95 Å, dark-salmon).^[9]^ The two Fe atoms are coordinated by six residues, including four glutamates and two histidines. Water molecules are displayed as marine spheres and are numbered W1 and W4 according to their positions. The coordination distances are depicted in gray, and the Fe–Fe distances are represented in red. The unit of distance is Ångström (Å).

**
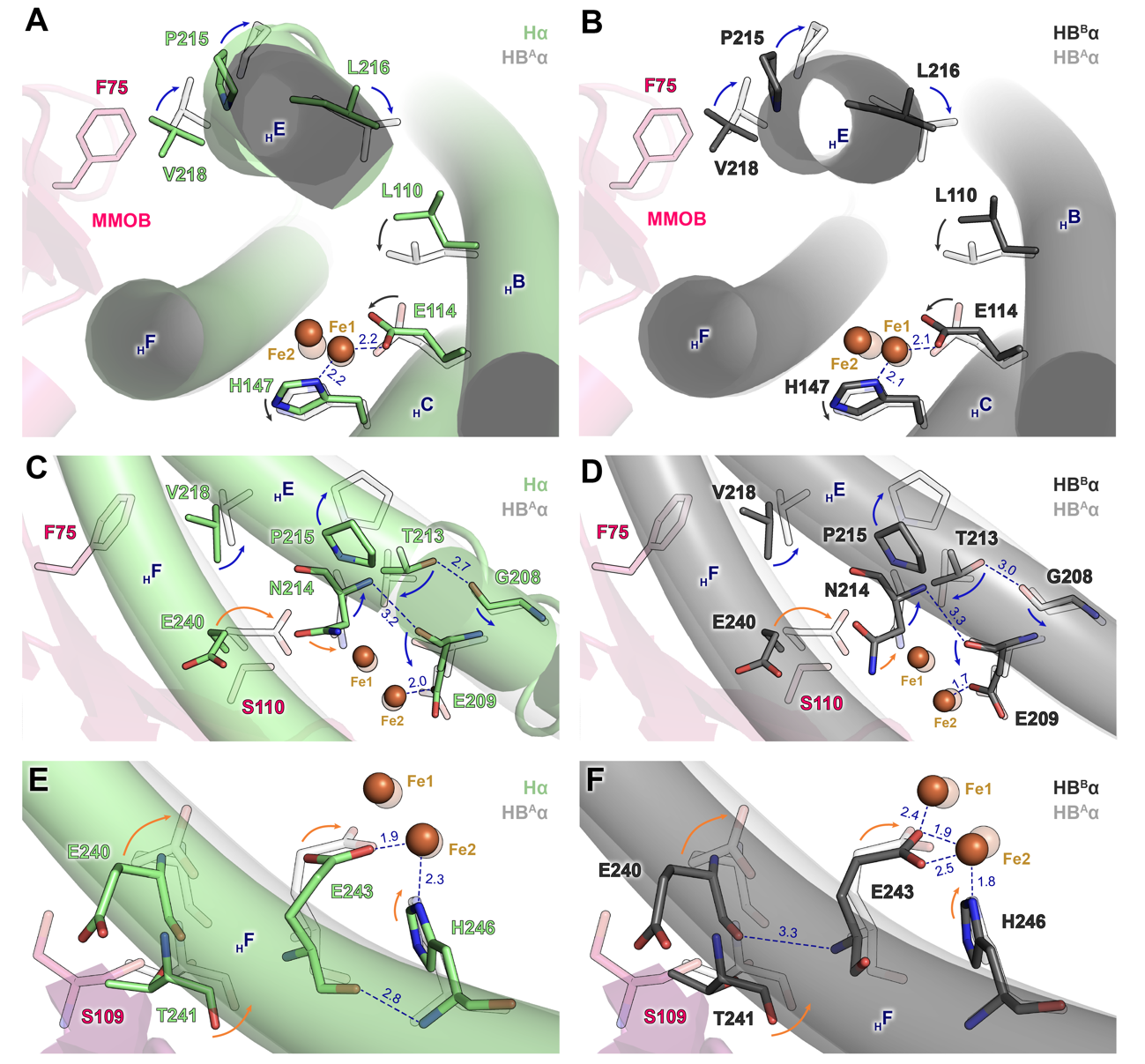
**

**Figure S12.** The key residues of the MMOH α-subunit related to the di-iron active site upon MMOB binding with cryo-electron microscopy (EM). (A and B) Structural alignment of HB^A^ with (A) MMOH and (B) HB^B^ in the four-helix bundle. MMOB Phe75 triggers a shift in the residues coordinating Fe1 within the four-helix bundle.^[9]^ (C and D) Structural alignment of HB^A^ with (C) MMOH and (D) HB^B^ in helices E and F, highlighting conformational changes related to the π-helix and Fe1.^[7,10]^ (E and F) Structural alignment of HB^A^ with (E) MMOH and (F) HB^B^ in helix F, focusing on residues involved in Fe2 coordination. The color of the arrows indicates conformational changes caused by NT-HB^A^β (blue) and the core region of MMOB (orange). Black arrows indicate conformational changes in the residues of helices B and C. The color of the dotted box is based on the color of the arrow indicating the conformational changes.

**
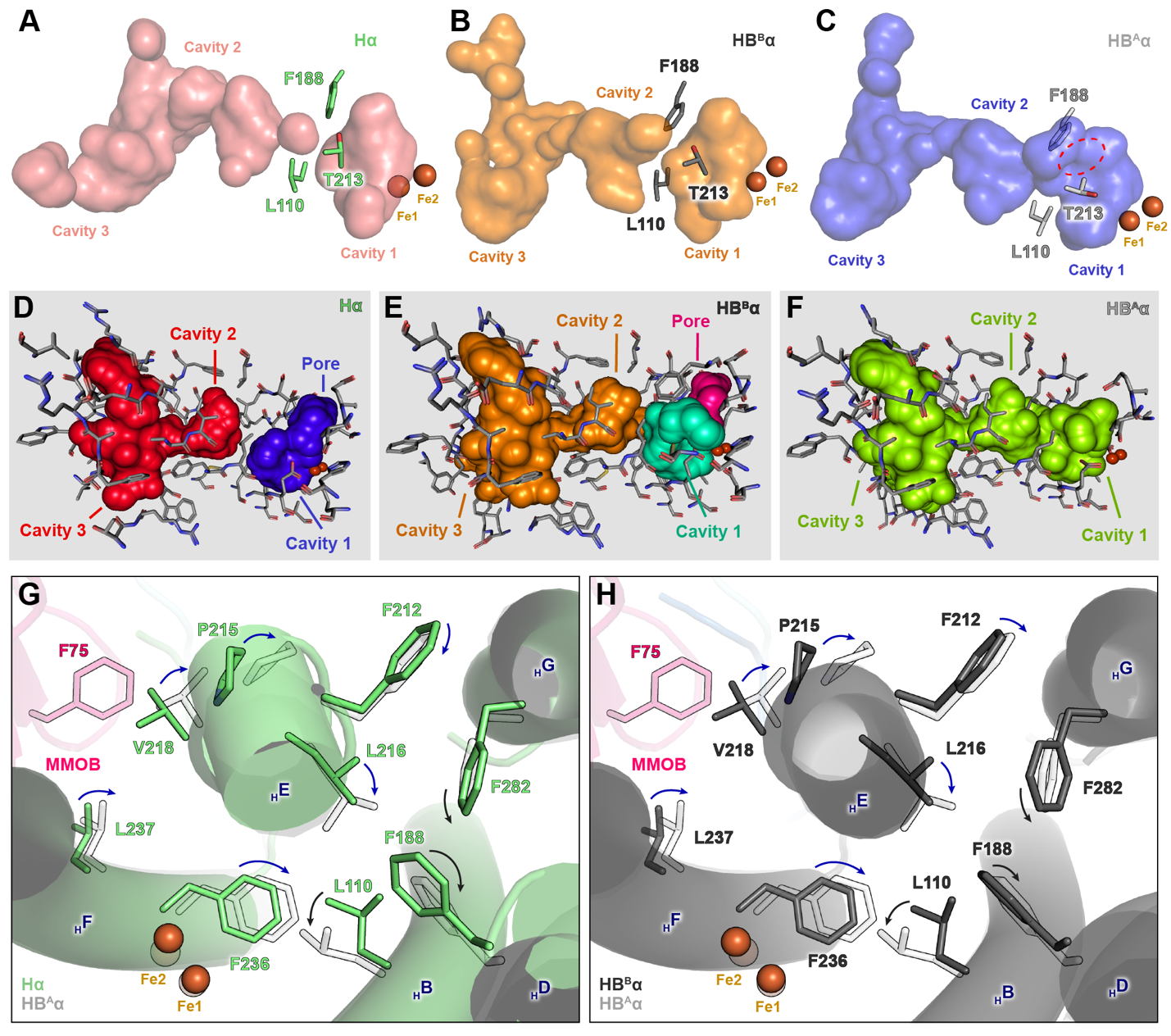
**

**Figure** **S13.** Analysis of cavities in cryo-EM structures of sMMO. The cavities were displayed using (A-C) PyMOL 2.5.2 and (D-F) CAVER Analyst 2.0 BETA.^[11]^ Views of cavities 1, 2, and 3 are shown as translucent van der Waals surfaces in the interior of (A) MMOH (residue, lime; surface, deep-salmon), (B) HB^B^ (residue, dark; surface, orange), and (C) HB^A^ (residue, gray; surface, tv_blue). The cavities were displayed using PyMOL 2.5.2 with the following parameters: display quality, maximum; surface, cavities and pockets (culled); cavity detection radius, three solvent radii; cavity detection cutoff, five solvent radii; and HETATMs were ignored. The cavities were calculated in the interior of (D) MMOH, (E) HB^B^, and (F) HB^A^ using CAVER Analyst 2.0 BETA with the following parameters: probe, 1.5 Å; large probe, 3.0 Å. (G-H) Conformational changes in residues related to cavities upon binding MMOB with cryo-EM. Structural alignment of HB^A^ with (G) MMOH and (H) HB^B^. Blue arrows indicate conformational changes caused by NT-HB^A^β. Black arrows indicate conformational changes in the residues from helices B, D, and G.

**
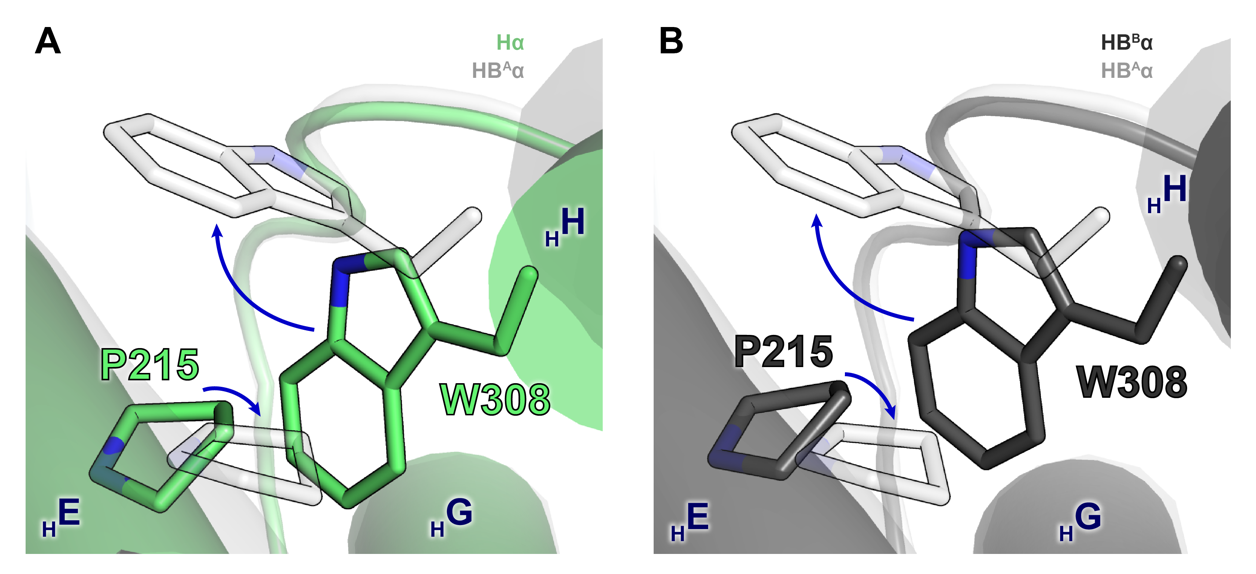
**

**Figure S14.** Conformational rearrangements of the proposed O_2_-entry tunnel gate residues in the cryo-EM H-1B structure. (A-B) Positions of Pro215 and Trp308 in (A) cryo-EM MMOH alone and (B) cryo-EM HB^B^. The blue arrows indicates conformational changes induced by MMOB binding.

**
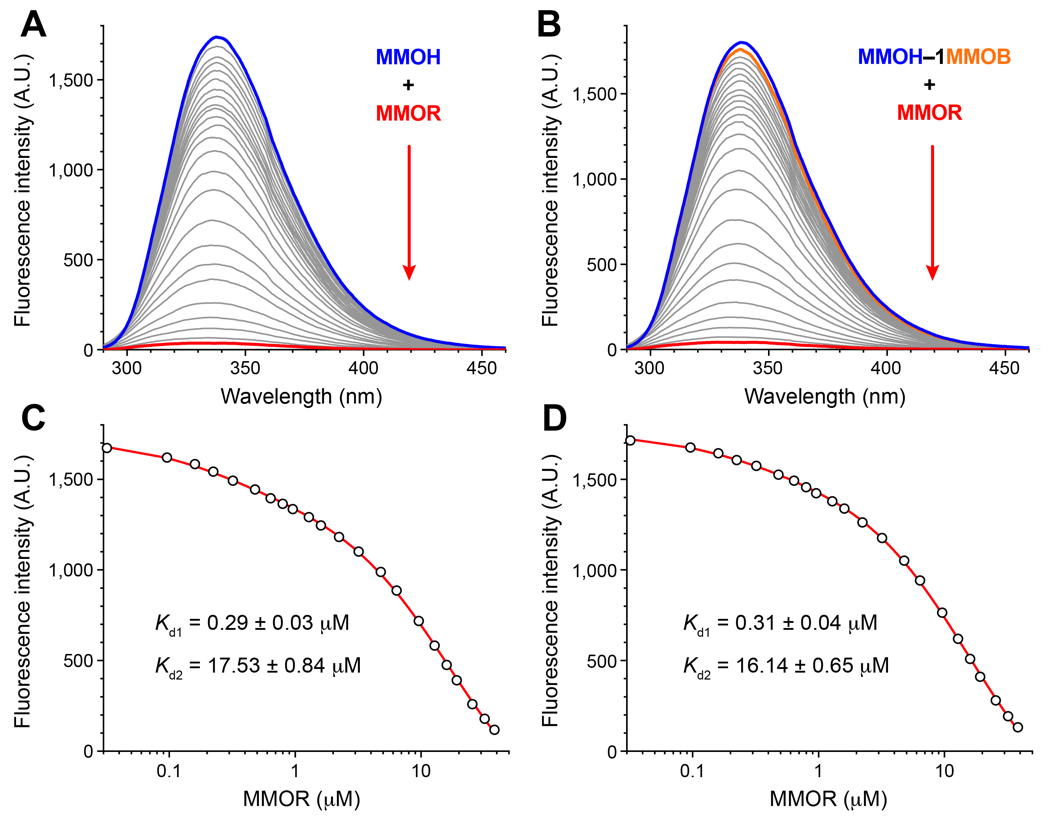
**

**Figure S15.** Titration of MMOR to MMOH in the presence and absence of MMOB. (A-B) Tryptophan quenching of MMOH upon the addition of MMOR without (A) or with (B) one equivalent MMOB. (C-D) Binding affinity between MMOH and MMOR without (C) or with (D) one equivalent MMOB. All titrations were performed using oxidized MMOH and reduced MMOR. Reduced MMOR was prepared by mixing MMOR with an equimolar amount of NADH before measurement in N_2_ (g)-filled glovebox. The *K*_d_ value was calculated by curve fitting with the change in the fluorescence intensity at 336 nm. The x-axis is represented in log scale to show the distinct binding affinities.

**Table S1.** Cryo-EM data collection, refinement, and validation statistics.

|  | #1 MMOH  (EMDB-39540)  (PDB 8YRD) | #2 MMOH-1MMOB  (EMDB-38391)  (PDB 8XIW) |
| --- | --- | --- |
| **Data collection and processing** |  |  |
| Magnification | 105,000 | 105,000 |
| Voltage (kV) | 300 | 300 |
| Electron exposure (e^–^/Å^2^) | 65.4 | 58.2 |
| Defocus range (µm) | -0.8 to -2.3 | -0.9 to -2.3 |
| Pixel size (Å) | 0.848 | 0.849 |
| Symmetry imposed | C2 | C1 |
| Micrographs collected/used | 8,475/8,171 | 15,007/9,471 |
| Initial particle images (no.) | 407,859 | 200,043 |
| Final particle images (no.) | 4,779,030 | 7,714,850 |
| Map resolution (Å) | 2.64 | 2.85 |
| Unmasked resolution at 0.5/0.143 FSC (Å) | 2.6/2.9 | 3.2/2.8 |
| Masked resolution at 0.5/0.143 FSC (Å) | 2.8/2.5 | 3.1/2.7 |
|  |  |  |
| **Structural refinement** |  |  |
| Initial model used (PDB code) | 1MTY | 4GAM |
| Model composition |  |  |
| Chains | 9 | 10 |
| Non-hydrogen atoms | 17,147 | 18,397 |
| Protein residues | 2,108 | 2,266 |
| Ligands | 4 | 4 |
| Mean *B* factors (Å^2^) |  |  |
| Protein | 37.62 | 105.25 |
| Ligand | 47.98 | 120.74 |
| Water | 49.42 | 59.38 |
| R.m.s. deviations |  |  |
| Bond lengths (Å) (# > 4σ) | 0.005 (0) | 0.006 (0) |
| Bond angles (°) (# > 4σ) | 0.738 (0) | 1.053 (0) |
| Validation |  |  |
| Molprobity score | 0.86 | 0.69 |
| Clash score | 0.72 | 0.11 |
| Poor rotamers (%) | 0.92 | 0.86 |
| Cβ outliers (%) | 0.00 | 0.00 |
| CaBLAM outliers (%) | 0.29 | 0.63 |
| CC (mask) | 0.86 | 0.87 |
| Ramachandran plot |  |  |
| Favoured (%) | 97.33 | 97.25 |
| Allowed (%) | 2.67 | 2.75 |
| Disallowed (%) | 0.00 | 0.00 |

MMOH: methane monooxygenase hydroxylase
MMOB: MMO regulatory protein
FSC: Fourier Shell Correlation
CaBLAM: Cα-based low-resolution annotation method

**Table S2.** Metal coordination in the active sites of different forms of MMOH.

| Bond | Distance, Å | | | | | | | | | | | | | |
| --- | --- | --- | --- | --- | --- | --- | --- | --- | --- | --- | --- | --- | --- | --- |
|  | **Cyro-EM** | | | **X-ray crystallography** | | | | | | | | **XFEL** | | |
|  | *Methylosinus sporium* 5 | | | | *Methylococcus capsulatus* Bath | | | *Methylosinus trichosporium* OB3b | | | | | |  |
|  | H | HB^B^ | HB^A^ | H -2D | H_ox_ | H_red_ | H -2B | H_ox_ | H_red_ | H_ox_ -2B | H_red_ -2B | H_ox_ -2B | H_red_ -2B |  |
| Fe1-Fe2 | **3.1** | **3.1** | **2.7** | **3.1** | **2.9** | **3.3** | **2.9** | **3.1** | **3.3** | **3.5** | **3.4** | **3.0** | **3.3** |  |
| Fe1-E114(OE1) | 2.2 | 2.1 | 1.9 | 2.0 | 1.9 | 2.1 | 1.9 | 2.0 | 2.0 | 2.0 | 1.9 | 1.8 | 2.1 |  |
| Fe1-E114(OE2) | *^a^* | *^a^* | *^a^* | 2.4 | *^a^* | *^a^* | *^a^* | *^a^* | *^a^* | *^a^* | *^a^* | *^a^* | *^a^* |  |
| Fe1-E144(OE1) | 2.0 | 2.3 | 1.9 | 2.1 | 2.0 | 2.2 | 2.1 | 2.1 | 2.3 | 2.0 | 2.0 | 2.0 | 2.1 |  |
| Fe2-E144(OE2) | 2.2 | 2.4 | 2.1 | *^a^* | 2.4 | 2.3 | 2.2 | *2.2* | 2.2 | 2.1 | 2.1 | 2.1 | 2.1 |  |
| Fe1-H147 | 2.3 | 2.1 | 2.5 | *^a^* | 2.1 | 2.3 | 2.1 | *2.2* | 2.2 | 2.2 | 2.1 | 2.3 | 2.2 |  |
| Fe2-E209 | 2.0 | 1.7 | 1.7 | 1.9 | 2.0 | 2.0 | 2.0 | 1.9 | 2.0 | 2.1 | 2.1 | 2.0 | 2.2 |  |
| Fe1-E243(OE1) | *^a^* | 2.4 | 2.2 | *^a^* | *^a^* | 2.4 | 1.9 | *^a^* | 2.2 | *^a^* | *^a^* | *^a^* | 2.2 |  |
| Fe2-E243(OE1) | *^a^* | 1.9 | 2.5 | 2.3 | *^a^* | 2.5 | 2.1 | *^a^* | 2.4 | *^a^* | *^a^* | *^a^* | 2.4 |  |
| Fe2-E243(OE2) | 1.9 | 2.5 | 2.5 | 2.2 | 2.0 | 2.4 | 2.0 | 2.2 | 2.2 | 2.2 | 2.0 | 2.0 | 2.2 |  |
| Fe2-H246 | 2.3 | 1.8 | 2.0 | 2.2 | 2.2 | 2.2 | 2.5 | 2.2 | 2.2 | 2.3 | 2.3 | 2.2 | 2.2 |  |
| W1-E114(OE2) | 2.7 | 2.7 | *^a^* | *^a^* | 2.9 | 2.6 | *^a^* | 2.2 | 2.8 | 2.0 | 2.0 | 2.5 | 2.4 |  |
| W2-E114(OE2) | *^a^* | *^a^* | 2.6 | *^a^* | *^a^* | *^a^* | *^a^* | *^a^* | *^a^* | *^a^* | *^a^* | *^a^* | *^a^* |  |
| W1-E243(OE1) | 3.5 | 2.9 | 2.5 | *^a^* | 2.7 | 3.1 | *^a^* | 3.1 | *^a^* | 3.0 | 2.7 | 2.7 | 2.9 |  |
| W1-Fe1 | 2.1 | 2.3 | 2.2 | *^a^* | 2.3 | 2.5 | *^a^* | 2.2 | 2.4 | 2.0 | 2.0 | 2.1 | 2.1 |  |
| W2-Fe2 | 3.9 | 4.0 | 4.0 | *^a^* | *^a^* | *^a^* | 3.5 | *^a^* | *^a^* | *^a^* | *^a^* | *^a^* | *^a^* |  |
| W3-Fe1 | *^a^* | *^a^* | *^a^* | *^a^* | *^a^* | 2.4 | *^a^* | *^a^* | 2.5 | *^a^* | *^a^* | *^a^* | 2.4 |  |
| W3-Fe2 | *^a^* | *^a^* | *^a^* | *^a^* | *^a^* | 2.7 | *^a^* | *^a^* | 2.3 | *^a^* | *^a^* | *^a^* | 2.5 |  |
| W4-Fe1 | *^a^* | *^a^* | *^a^* | 3.2 | *^a^* | *^a^* | *^a^* | *^a^* | *^a^* | *^a^* | *^a^* | *^a^* | *^a^* |  |
| W4-H147 | *^a^* | *^a^* | *^a^* | 3.0 | *^a^* | *^a^* | *^a^* | *^a^* | *^a^* | *^a^* | *^a^* | *^a^* | *^a^* |  |
| Fe1-OH^-^ | *^a^* | *^a^* | *^a^* | *^a^* | 1.8/2.4 | *^a^* | *^a^* | 2.0 | *^a^* | 1.9 | 1.8 | 2.0/2.2 | *^a^* |  |
| Fe2-OH^-^ | *^a^* | *^a^* | *^a^* | *^a^* | 1.9/2.6 | *^a^* | *^a^* | 1.9 | *^a^* | 2.0 | 1.9 | 2.1/2.2 | *^a^* |  |

*^a^*Not applicable
MMOH: methane monooxygenase hydroxylase
Cryo-EM: cryogenic electron microscopy
XFEL: X-ray free-electron laser

**Video S1.** Structural change of MMOH induced by MMOB with 3D variability analysis (front view).^[1]^

**Video S2.** Structural change of MMOH induced by MMOB with 3D variability analysis (top view).

**Video S3.** Structural change of MMOH induced by MMOB with 3D variability analysis (side view).

**Video S4.** Monitoring the motion of MMOH through 3D flexible refinement (front view).^[12]^

**Video S5.** Monitoring the motion of MMOH through 3D flexible refinement (top view).

**Video S6.** Monitoring the motion of MMOH through 3D flexible refinement (side view).

**Video S7.** MMOB–induced structural dynamics of the di-iron active site revealed by 3D variability analysis.

**Video S8.** MMOB-induced structural dynamics of residues involved in cavities 1–2 connectivity revealed by 3D variability analysis.

**References**

[1] A. Punjani, D. J. Fleet, *J. Struct. Biol.* **2021**, *213*, 107702.

[2] S. J. Lee, M. S. McCormick, S. J. Lippard, U.-S. Cho, *Nature* **2013**, *494*, 380-384.

[3] A. C. Rosenzweig, C. A. Frederick, S. J. Lippard, P. Nordlund, auml, *Nature* **1993**, *366*, 537-543.

[4] E. Roberts, J. Eargle, D. Wright, Z. Luthey-Schulten, *BMC Bioinf.* **2006**, *7*, 1-11.

[5] H. Kim, S. An, Y. R. Park, H. Jang, H. Yoo, S. H. Park, S. J. Lee, U.-S. Cho, *Sci. Adv.* **2019**, *5*, eaax0059.

[6] A. C. Rosenzweig, H. Brandstetter, D. A. Whittington, P. Nordlund, S. J. Lippard, C. A. Frederick, *Proteins: Struct., Funct., Bioinf.* **1997**, *29*, 141-152.

[7] J. C. Jones, R. Banerjee, K. Shi, H. Aihara, J. D. Lipscomb, *Biochemistry* **2020**, *59*, 2946-2961.

[8] D. A. Whittington, S. J. Lippard, *J. Am. Chem. Soc.* **2001**, *123*, 827-838.

[9] V. Srinivas, R. Banerjee, H. Lebrette, J. C. Jones, O. Aurelius, I.-S. Kim, C. C. Pham, S. Gul, K. D. Sutherlin, A. Bhowmick, *J. Am. Chem. Soc.* **2020**, *142*, 14249-14266.

[10] D. Rinaldo, D. M. Philipp, S. J. Lippard, R. A. Friesner, *J. Am. Chem. Soc.* **2007**, *129*, 3135-3147.

[11] A. Jurcik, D. Bednar, J. Byska, S. M. Marques, K. Furmanova, L. Daniel, P. Kokkonen, J. Brezovsky, O. Strnad, J. Stourac, *Bioinformatics* **2018**, *34*, 3586-3588.

[12] A. Punjani, D. J. Fleet, *Nat. Methods* **2023**, *20*, 860-870.
